# Unveiling Supramolecular Structures Formed by Menthol and Xanthan Gum in Oleic Acid-Based Microemulsions

**DOI:** 10.1021/acsomega.5c10561

**Published:** 2026-01-29

**Authors:** Rafael Leonne Cruz de Jesus, Letícia Maria Silva Amaral, Tainá Santos Souza, Guilherme A. Ferreira, Bruna Aparecida Souza Machado, Diogo Rodrigo Magalhães Moreira, Henrique Rodrigues Marcelino, Darizy Flávia Silva

**Affiliations:** † Graduation Program in Pharmacy, College of Pharmacy, 28111 Federal University of Bahia, Salvador, Bahia 40170-115, Brazil; ‡ Department of Medicines, College of Pharmacy, Federal University of Bahia, Salvador, Bahia 40170-115, Brazil; § Department of Physical Chemistry, Institute of Chemistry, 28111 Federal University of Bahia, Salvador, Bahia 40170-115, Brazil; ∥ SENAI Institute of Innovation (ISI) in Health Advanced Systems (CIMATEC ISI SAS), University Center SENAI/CIMATEC, Salvador 41650-010, Brazil; ⊥ Fundação Oswaldo Cruz, Instituto Gonçalo Moniz, Salvador, Bahia CEP 40296-710, Brazil; # Department of Bioregulation, Institute Health Sciences, Federal University of Bahia, Salvador, Bahia 41100-110, Brazil

## Abstract

Microemulsions (MEs) are delivery systems that can enhance the rate of drug dissolution due to their supramolecular structure. However, adding drugs, such as menthol, and thickness agents, like xanthan gum (XG), can significantly modify these nanoscaled architectures. This study aimed to understand the mechanistic basis of supramolecular structural modifications in oleic acid-based MEs induced by XG and menthol addition. A multitechnique approach combining electron microscopy, dynamic light scattering (DLS), small-angle X-ray scattering (SAXS), rheology, and texture analysis was employed to characterize formulations containing concentration ranges of menthol (0.1–1.0%w/w) and XG (0.1–0.5%w/w). This complementary analytical strategy enabled the detection of structural changes across different length scales. Our findings revealed that menthol addition leads to droplet diameter modulation (DLS: 127–157 nm; SAXS: 108–136 nm), suggesting drug entrapment within the oil phase. XG addition produces a distinctive interconnected “pearl-necklace” supramolecular architecture without significant droplet size changes (DLS: 102–111 nm; SAXS: 102 nm). Crucially, rheological and textural analyses detected physical property modifications not identified by light scattering techniques, with XG dramatically influencing consistency, firmness, and adhesiveness, while menthol enhanced adhesive properties. These structure–function correlations showed that rational ME design can be achieved through controlled excipient addition. Collectively, the nanoscaled pearl-necklace architecture and enhanced adhesive properties suggest promising applications for sustained topical drug delivery, warranting future investigation of skin permeation kinetics and therapeutic efficacy in dermatological formulations.

## Graphical abstract

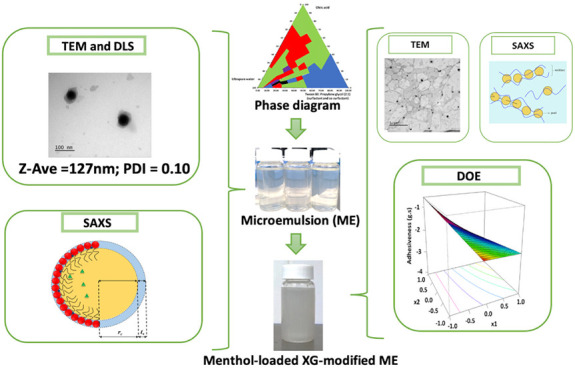

## 1 Introduction

Microemulsions (MEs) are thermodynamically stable, isotropic mixtures composed of oil, water, surfactants, and very often, of cosurfactants.^1^ These systems represent a unique class of soft matter where interfacial tension approaches ultralow values (∼10^–3^ mN/m), enabling spontaneous emulsification and long-term thermodynamic stability.^2^ The distinctive supramolecular architecture of MEs, governed by critical packing parameters and interfacial curvature, allows the solubilization of lipophilic compounds while maintaining optical clarity and low viscosity. Thus, gaining considerable attention across the pharmaceutical, cosmetic, and food industries.^2^ Their application in drug delivery is particularly notable: MEs can improve the bioavailability of poorly water-soluble compounds.^1,3,4^ For instance, the reformulation of cyclosporine in Neoral significantly enhanced its pharmacokinetic profile compared to Sandimmune by minimizing dependence on bile and food for absorption,^5,6^ a milestone that underscored the therapeutic value of MEs for drugs with narrow therapeutic windows.

Despite the advantages described above, any conventional fluid MEs can very often have a relatively low viscosity, which limits their application in topical and dermatological formulations.^7,8^ Low-viscosity systems are prone to rapid runoff and poor skin retention, reducing therapeutic efficacy.^9^ To overcome these drawbacks, adding biopolymers as rheology modifiers has emerged as an effective strategy to increase viscosity and structural stability without compromising the balance of interfacial forces while modulating viscoelastic properties.^10^ Here, a key challenge lies in achieving viscosity enhancement without disrupting the thermodynamic stability or nanoscale organization that defines MEs properties. Among the most common rheology modifiers, xanthan gum (XG) stands out for its biocompatibility, biodegradability, low cost, and regulatory agencies’ approval (e.g., FDA) for pharmaceutical use.^11–14^

XG, a microbial exopolysaccharide produced by *Xanthomonas campestris*, exhibits pronounced shear-thinning behavior and high thickening capacity even at low concentrations.^15,16^ These properties make it a valuable excipient in semisolid systems for controlled drug release and prolonged topical retention.^17^ Furthermore, XG provides mucoadhesive characteristics and modulates the mechanical behavior of formulations. Its interaction with emulsified systems leads to pseudoplastic rheology, stabilizes oil droplets by increasing continuous-phase viscosity, and improves formulation spreadability. Beyond bulk rheological modification, XG potentially influences interfacial properties through competitive adsorption at oil–water interfaces, which may alter droplet stabilization mechanisms and create novel supramolecular architectures.^11,17,18^

The current understanding of XG-MEs interactions remains phenomenological, focusing primarily on macroscopic rheological characterization while overlooking critical nanoscale structural modifications. For instance, Bobade et al. (2018) and Banaś and Harasym (2021) showed the non-Newtonian behavior and Oleogel-stabilizing ability,^19,20^ while Jadav et al. (2023) highlighted XG biocompatibility in sustained-release formulations.^21^ Koop et al. (2012) demonstrated improved topical delivery of curcumin using xanthan–galactomannan gels.^22^ Djekic et al. (2016) reported six-month stability of XG-based MEs with sustained ibuprofen release and in vivo efficacy,^17^ and Mishra et al. (2018) showed enhanced liranaftate permeability in XG MEs.^23^ However, a fundamental question remains: does XG increase continuous-phase viscosity or induce structural transitions that create new interfacial architectures? This distinction is crucial for rational formulation design and directly impacts drug release kinetics and permeation enhancement.

In addition to biopolymers, active components such as menthol play a crucial role in the structure and its release from the drug delivery system. Menthol, a natural monoterpene alcohol, is prized for its permeation-enhancing effects but is challenged by low water solubility.^24–26^ When loaded into MEs, it can alter droplet size and phase behavior.^27,28^ Some studies report menthol partitioning into the oil core (increasing droplet size by DLS), but this technique cannot distinguish between actual size increases, interfacial reorganization, or formation of nonspherical structures.

To addressing some of these gaps, this present study has employed a comprehensive multiscale characterization approach to explore the physicochemical and supramolecular characteristics of oleic acid–based MEs thickened with XG and incorporating menthol. Employing complementary techniques—including small-angle X-ray scattering (SAXS) to elucidate nanoscale structuring, transmission electron microscopy (TEM) for direct visualization, rheological profiling to assess flow behavior, and texture profile analysis to evaluate mechanical properties, we aim to (i) elucidate the molecular-level distribution and structural impact of menthol incorporation using complementary scattering and microscopy techniques; (ii) determine how XG modifies ME interfacial architecture and creates potential network structures through systematic concentration studies; and (iii) establish quantitative structure–property relationships linking nanoscale organization to macroscopic functional performance.

By integrating this multiscale analytical approach with a factorial experimental design and correlation analyses, we seek to provide fundamental insights into biopolymer-modified ME systems that enable rational design of next-generation topical formulations. Ultimately, this work addresses a critical gap in soft matter science by connecting molecular-level interactions to interfacial phenomena and ultimately to macroscopic performance—a multiscale understanding essential for advancing colloidal drug delivery systems.

## 2 Experimental Section

### 2.1 Materials

Oleic acid and propylene glycol were purchased from Labsynth (Synth, Diadema/SP, Brazil). Tween 80 and XG were obtained from Êxodo Scientific (Êxodo, Sumaré/SP, Brazil). Menthol was purchased from Dinâmica Química Contemporânea LTDA (Dinâmica, São Paulo, Brazil). Ultrapure water (Milli-Q, Millipore) was used. All other raw materials were analytical grade.

### 2.2 Pseudoternary Phase Diagram

Pseudoternary phase diagrams were constructed using the water titration method.^29,30^ The mixture was prepared in the first step of each experiment with surfactant/cosurfactant (Tween 80/propylene glycol). Tween 80 was chosen because it contains oleic acid on its composition, which can pave the way for intermolecular interactions with the oil. The weight ratio of these components was kept constant in each experiment. Afterward, the oil (oleic acid) phase was added to the obtained surfactant/cosurfactant mixture at the weight ratios of 1:9, 2:8, 3:7, 4:6, 5:5, 6:4, 7:3, 8:2, and 9:1 w/w. Each sample was titrated with water (first point: 8% of total amount and final point equal to 88% of total content) and sonicated with a digital ultrasonic cell disruptor (Sonicator 500W, UNIQUE, São Paulo, Brazil) for 5 min. Fully transparent, one-phase liquids were classified as ME.

### 2.3 Microemulsion Preparation

A specific formulation was prepared, within the ratio where the dispersion on the pseudoternary diagram was translucent, fluid, and homogeneous (Supp Figure S1). Thus, a formulation (ME) containing oleic acid (4.0%w/w), propylene glycol (4.4%w/w), Tween 80 (8.6%w/w), and water (83%w/w) was prepared. The preparation process involved sonication (500 W, UNIQUE, São Paulo, Brazil) 40% amplitude, for 5 cycles of 5 min followed by 3 min of rest on the bench at room temperature.

For the formulations containing menthol, the molecule was added to the formulation through magnetic stirring for 2 h, to achieve final menthol concentrations of 0.1% w/w (ME + menthol 0.1%) or 1.0% w/w (ME + menthol 1.0%). All formulations (both loaded and unloaded) were stored for 24 h to allow the formulations to reach equilibrium.

### 2.4 Stability Studies

To evaluate the influence of temperature and menthol on the stability of the formulation, the ME, ME + menthol 0.1% and ME + menthol 1.0% were systematically monitored over 90 days, with analyses conducted on days 1 (24 h later preparation), 7, 15, 30, 60, and 90 under storage temperatures of 5 °C (refrigerator) and 30 °C (oven). Throughout this period, evaluations included examinations of color, odor, visual phase separation, and measurements of pH, conductivity, and turbidity. For each tested condition, three formulation samples, each weighing 20 g, were used. The pH measurements were performed using a model PH-5000 pH meter (Digital Instruments, Bresso, Italy). Electrical conductivity was determined using a benchtop conductivity meter (W12D, BEL Engineering, Monza, Italy). Turbidity analysis was performed using a portable digital turbidimeter (Del Lab, model DLT-WV, São Paulo, Brazil), with a measurement range of 0 to 1000 NTU (Nephelometric Turbidity Units). All tests were performed in triplicate at 25.0 ± 0.5 °C, and the mean and standard deviation were calculated.

### 2.5 Preparation of Menthol-Loaded XG-Modified ME (men-XG-ME)

The ME was prepared according to the previously described procedure. Menthol was then weighed and solubilized in 20g of ME using a magnetic stirrer for 2 h. Here, the 0.05, 0.1, or 0.15%w/w menthol concentrations were evaluated. After this time, XG was weighed and mixed in menthol-loaded ME for 20 h at room temperature using a magnetic stirrer. The compositions of the men-XG-ME samples are shown in Table 1.

**Table 1:** Composition of the Menthol-Loaded XG-Modified ME (men-XG-ME)

	men-XG-ME1	men-XG-ME2	men-XG-ME3	men-XG-ME4	men-XG-ME 5, 6 and 7
ME (g)	20.0	20.0	20.0	20.0	20.0
Menthol (%)^a^	0.05	0.15	0.05	0.15	0.10
XG (%)^a^	0.10	0.10	0.50	0.50	0.30

a The concentration (% w/w) of menthol and XG refers to their concentration in a previously prepared 20 g ME formulation.

Finally, Response Surface Methodology (RSM), a specialized technique within the Design of Experiments (DOE), was employed to model the influence of the menthol and xanthan gum over some physical and physicochemical properties.

### 2.6 Macroscopic Characterization

A visual inspection was performed to assess macroscopic properties, including color, consistency, and homogeneity.

### 2.7 Transmission Electron Microscopy

The morphology of MEs (ME, ME + menthol 0.1% and ME + menthol 1.0%) or men-XG-MEs (Table 1) droplets was examined using transmission electron microscopy (TEM) on a JEOL 1230 microscope (JEOL, Tokyo, Japan). For visualization, 10 μL of the formulations (ME, ME + menthol 0.1%, and ME + menthol 1.0%) were deposited onto Formvar-coated nickel grids and allowed to adsorb for 2 min. Excess sample was removed, and the grids were negatively stained with 0.5% phosphotungstic acid (1:1 v/v; sample:stain) for 30 s.

In a separate set of experiments, men-XG-ME1 to ME5 formulations (Table 1) were analyzed. Due to their high viscosity, these samples were initially diluted (1:10 v/v) with water before grid preparation. Ten μL of the diluted dispersions were then applied to Formvar-coated nickel grids, allowed to adsorb for 2 min, and negatively stained with 0.5% phosphotungstic acid (1:1 v/v) for 30 s.

All grids were subsequently stored in a closed container for 24 h to allow complete evaporation of residual water before imaging. TEM observations were carried out at an accelerating voltage of 15 kV.

### 2.8 Dynamic Laser Scattering (DLS)

The mean droplet size, expressed as the Z-average hydrodynamic diameter, and the polydispersity index (PDI) of MEs, either containing or not menthol (0.1 or 1.0% w/w), as well as formulations men-XG-ME1 to ME7, were determined by dynamic light scattering (DLS) using a Zetasizer (Malvern Instruments, UK). Prior to measurement, samples were diluted (1:150, v/v) with ultrapure Milli-Q water (Millipore, Australia) to ensure that the scattering intensity remained within the optimal detection range of the instrument. Measurements were carried out at a fixed scattering angle of 90° and a controlled temperature of 25 ± 1 °C. Each formulation was analyzed in triplicate, and mean values were considered for subsequent analysis.

### 2.9 Small Angle X-ray Scattering (SAXS) Measurements

SAXS measurements were performed using a pinhole camera (MolMet, Rigaku, Japan, modified by SAXSLAB/Xenocs) connected to a microfocused X-ray generator (Rigaku MicroMax 003) with a Pilatus 300 K detector. The samples were analyzed in fused silica capillaries (Hilgenberg) with a diameter of 1.3 mm at 25 °C. The 2D images obtained were integrated and processed with Fit2D software to obtain the 1D scattering function *I*(*q*), where q=(4πλ)sin(θ/2) , with λ being the wavelength (0.154 nm) and θ the scattering angle. The *I*(*q*) values were brought to an absolute scale by subtracting the solvent scattering intensity from the total scattering intensity and normalizing with toluene as reference.

The scattering functions obtained by SAXS can be described as *I*(*q*) = *nP*(*q*)*S*(*q*), where *n* is the number density of scattering objects (the ME droplets in this case), *P*(*q*) is the form factor of the droplets, which is related to their size and shape, and *S*(*q*) is the structure factor, related to the interdroplet interactions. In this work, the SASView software (http://www.sasview.org/) has been used to fit the SAXS data obtained for the three different formulations (ME, ME + menthol 0.1%, and ME + menthol 1.0%) to the core–shell sphere model,^31,32^ while for selected formulations containing XG (Table 1), data were fitted to the pearl-necklace model.^33^ Detailed information regarding the models employed is presented in Supporting Information.

### 2.10 Rheological Profile

All formulations’ continuous flow and viscoelastic properties were analyzed on a rheometer with controlled stress (RN 2.1, Rheotest, Germany). The measurements were performed at 25 ± 0.5 °C. Before the experiments, all samples were equilibrated on the plate for 5 min to reach the desired temperature. The shear rate was increased linearly from 2 to 1000 s^–1^ and back, with each step lasting 20 s. Rheological measurements were performed on the samples listed in Table 1.

The consistency and flow indices of the formulations were determined using the Ostwald de-Waele model (eq 1).

1 $$\tau =k\times \gamma^{n}$$

where “τ” is the shear stress, “*k*” is the consistency index, “*γ*” is the shear rate, and “*n*” is the flow index.

### 2.11 Experimental Design and Response Surface Methodology (RSM) Modeling

To evaluate the influence of the concentrations of XG (X1) and menthol (X2) at two levels (factorial 2^2^ with a central point) and the responses (dependent variables) texture analysis, rheology, and other physicochemical properties was evaluated. Response Surface Methodology (RSM), a specialized technique within Design of Experiments (DOE), was employed to model and optimize the intricate relationships among multiple variables, facilitating the optimization of the data.^34^ All modeling and data analysis were performed using R software (version 4.1.0) with the packages rsm, olsrr, FrF2, ggplot2, ggpubr, and pid loaded and ruining at RStudio (version 1.4.1717) (Boston, MA, USA). Statistical significance was set at *p* < 0.05. The code used is available in the Supporting Information. GraphPad Prism© software, version 9.0 (GraphPad Software Inc., La Jolla, CA, USA) was also used for some graphics.

### 2.12 Texture Profile Analysis (TPA)

Using a TA.XT Express texture analyzer (Stable Micro Systems, Surrey, United Kingdom), men-XG-ME formulations’ TPA were evaluated by firmness, consistency, adhesiveness, and cohesiveness. The study employed a straightforward compression mode, wherein the men-XG-ME was placed within a 20g scintillation vial. For the compression test, a P/0.5R probe (1/2″ Cylinder Delrin) was utilized, with the probe initially penetrating the product at a speed of 1.0 mm/s before sample insertion, maintaining a velocity of 1.0 mm/s during the test and concluding with a speed of 10.0 mm/s after the test. The probe penetrated the product at a depth of 5.0 mm with a force sensitivity of 1.0 g and utilized a load cell capacity of 10.0 g.^35^ As depicted in Figure 1, firmness was ascertained by identifying the peak of the positive curve, consistency was quantified by calculating the area beneath the positive curve, cohesiveness was determined by the peak value of the negative curve, and the adhesiveness was derived from the area the negative curve.^36,37^ The TPA was performed with the samples listed in Table 1.

**Figure 1:**
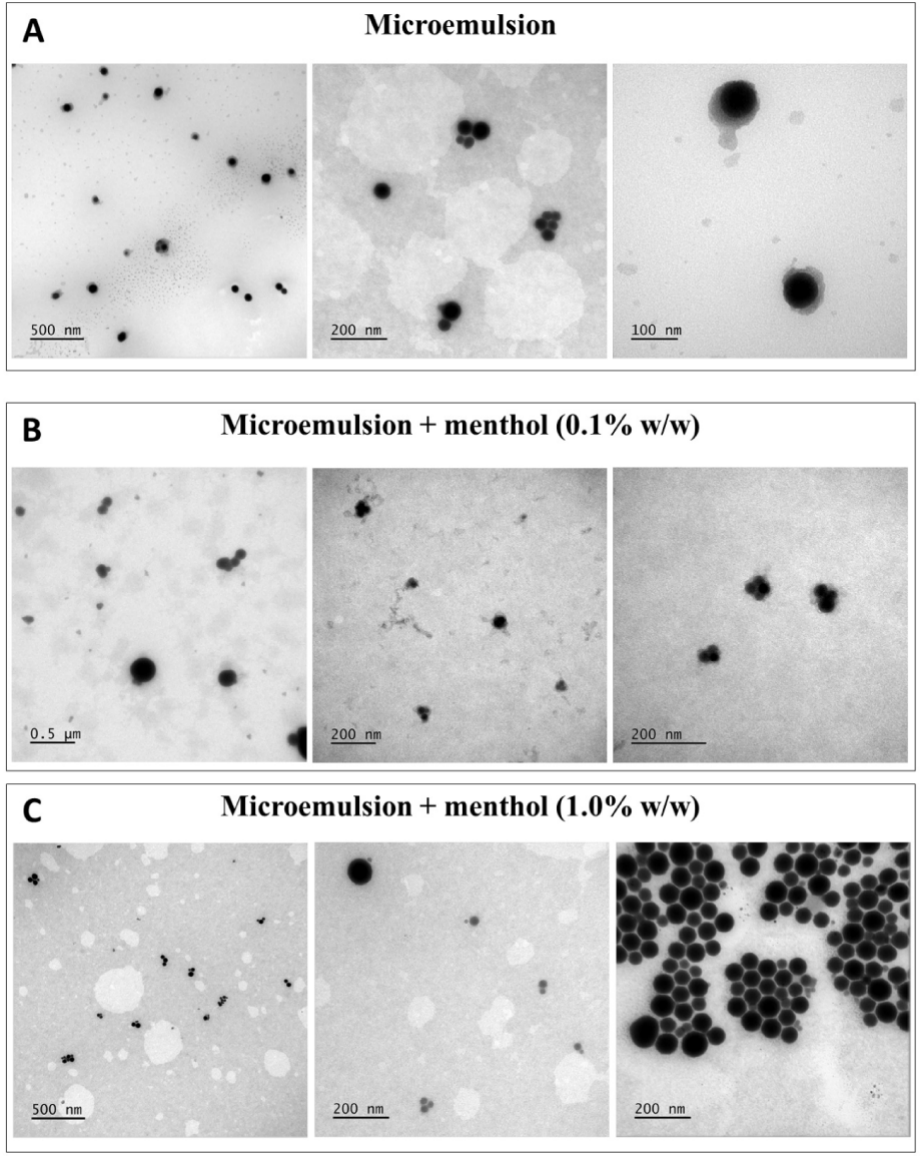
TEM images of ME droplets. (A) ME, (B) ME + menthol (0.1% w/w), and (C). ME + menthol (1.0% w/w). Scale bars: 500, 200, and 100 nm.

### 2.13 Statistical Analysis

GraphPad Prism^©^ software, version 9.0 (GraphPad Software Inc., La Jolla, CA, USA), was used for statistical analysis. Quantitative data were expressed as mean ± SD, and statistical analyses included a one-way analysis of variance (ANOVA) followed by a Tukey posttest. Pearson correlation coefficients between rheological and textural data were also calculated to detect significant differences (*p* < 0.05) between groups. Modeling and data analysis were performed using R software (version 4.1.0) and RStudio (version 1.4.1717) (Boston, MA, USA).

## 3 Results and Discussion

### 3.1 Characterization of Fluid Menthol-Loaded ME

As expected from the pseudoternary phase diagram (Supp Figure 1A), when prepared, the selected formulation exhibited the typical characteristics of a Winsor IV system^1,38^ (Supp Figure 1A). These findings reinforce the feasibility of the adopted methodology for developing stable mixtures of oil, surfactant (and/or cosurfactant), and water. Comparable macroscopic properties were also observed in formulations containing different concentrations of menthol (0.1% w/w and 1.0% w/w), although a slight increase in turbidity was detected in the formulation containing 1.0% menthol (Supp Figure 1B). This effect was further investigated by DLS and SAXS, which will be discussed later. Significantly, menthol addition did not compromise the macroscopic homogeneity or stability of the systems, as no phase separation or precipitation was observed during preparation or storage.

TEM was employed to investigate the morphology of the droplets in the MEs (ME, ME + menthol 0.1% and ME + menthol 1.0%).^39^ The micrographs revealed spherical droplets across all formulations, indicating the presence of discrete droplets dispersed within the continuous phase, consistent with a typical ME-like structure (Figure 1). Varying menthol concentrations did not affect droplet morphology. However, an increase in the mean droplet size was observed, as will be discussed later.

Interestingly, when samples were prepared with 2% phosphotungstic acid (PTA) and subjected to 48 h of drying (Supp Figure 2), both menthol-containing and menthol-free MEs displayed slightly elongated or irregular droplet morphologies. These alterations are likely artifacts resulting from evaporation-induced effects during TEM sample preparation. However, such morphological changes were not observed in samples prepared with 0.5% phosphotungstic acid and analyzed after 24 h of drying (Figure 1), corroborating the hypothesis that these artifacts and highlighting the importance of standardized preparation conditions for reliable TEM analysis.

Although TEM is a valuable tool for visualizing the morphology of colloidal systems, such as MEs, it has inherent limitations, particularly due to the drying process required for sample preparation. This procedure can introduce artifacts such as morphological deformation, droplet coalescence, or even structural disruption. To overcome these limitations and validate the morphological observations, complementary techniques including DLS and SAXS were employed, enabling the characterization of the MEs (ME, ME + menthol 0.1% and ME + menthol 1.0%) in their native hydrated state.

Our data from DLS measurements show that the ME formulation’s average droplet size was 127 ± 6 nm with a PDI = 0.11 ± 0.02 (Table 2). In addition, adding 0.1% or 1.0% menthol to ME increased the average droplet size from 145 ± 10 to 157 ± 6 nm, respectively. On the other hand, the PDI values remained approximately unchanged (0.10 ± 0.02 and 0.11 ± 0.02, for ME, ME + menthol 0.1% and ME + menthol 1.0%, respectively), indicating a negligible effect of menthol on the polydispersity index.

**Table 2:** Z-Average (nm) and Polydispersity Index (PDI) Determined by DLS, from ME^a^

Formulations	Z-Ave (nm)	PDI
ME	127 ± 6	0.11 ± 0.02
ME+menthol 0.1% w/w	145 ± 10	0.10 ± 0.02
ME+menthol 1.0% w/w	157 ± 6^b^	0.11 ± 0.02

a Data are given as average ± SD (*n* = 3).

b One-way ANOVA followed by Tukey posttest. (**p < 0.01 vs ME).

Previous studies indicated that adding menthol (1.0, 2.5, or 5.0%) in an ME may also increase the average droplet size compared to the unloaded ME.^27,28^ It is likely that the menthol, due to its hydrophobicity, can diffuse through the permeable film of surfactants and dissolve in the oily core of the droplet, leading to an increase in average size. Thus, for later studies looking for dermatological use of menthol-loaded MEs, attention should be given to the influence of droplet size on their application and their permeation through the skin. The smaller the droplets, the more probable it is for the ME to carry drugs to reach deeper skin layers.^40,41^

The SAXS curves for the studied MEs (ME, ME + menthol 0.1% w/w or ME + menthol 1.0% w/w) are shown in Figure 2A. The dots represent the experimental data, while the lines are the fits for the core–shell-sphere model, as illustrated in Figure 2B for a single ME droplet. As noticed, the theoretical model fits the data points for all formulations. These results indicate that the incorporation of menthol into ME does not cause changes in the droplets’ shape, i.e., morphology, consistent with the previously discussed results from TEM. A previous report has shown that incorporating compounds in ME can lead to changes in droplet morphology, e.g., a transition from spherical to cylindrical.^42^ Thus, while menthol did not cause changes at the levels tested in this study, other compounds or different concentrations could affect droplet morphology differently.

**Figure 2:**
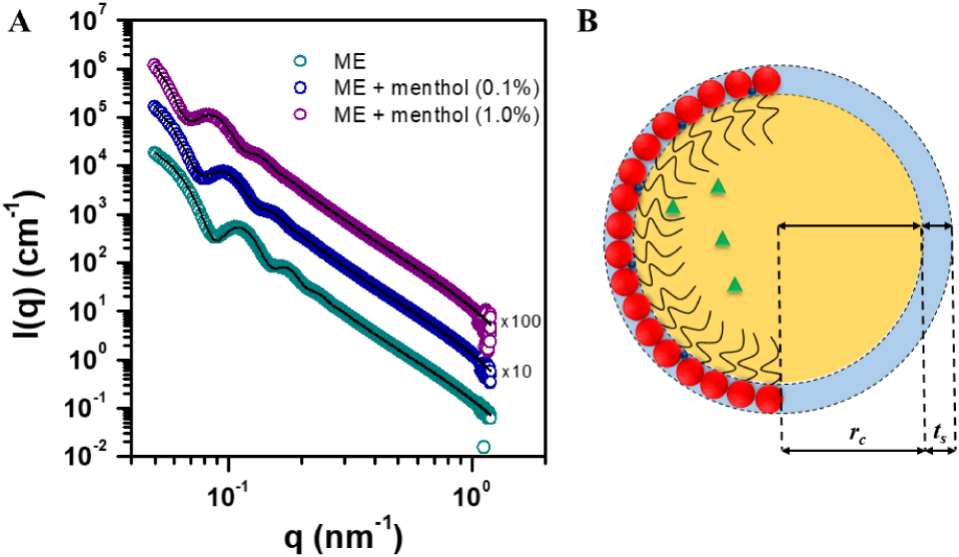
(A) SAXS curves (open dots) for the three studied ME and the fits (black lines) to the core–shell sphere model. The data are shifted along the *y*-axis for better visualization. (B) Schematic representation (not to scale) of the core–shell sphere model applied to a single ME droplet, with the corresponding dimensions resulting from the fits. The core is formed by the oil phase (yellow) and surfactant tails (black lines), while the shell consists of hydrated (light blue) polar surfactant head groups (red) and the cosurfactant (dark blue). Menthol (green) is dissolved in the oily core. The dimensions rc and ts represent the core radius and thickness of the shell, respectively.

Table 3 summarizes the dimensions of the ME droplets extracted from the mathematical models. The shell thickness (*t*_*s*_) can be calculated by subtracting the radius of the core (*r*_*c*_) from the total radius of the droplet (*r*_*s*_). The results show *t*_*s*_ values around 3.0 nm, consistent with the shell thickness previously reported for Tween 80 micelles in water.^43^ This observation indicates that the surfactant’s polar headgroup mainly controls this dimension, and the added cosurfactant effect is negligible at the concentrations tested. Furthermore, this shows that menthol is mainly located in the apolar region of ME, either between the surfactant tails and/or in the oleic acid core, confirming its hydrophobic character. This result is consistent with previous work describing that menthol has little or no effect on the hydrophilic shell of nonionic surfactant (Pluronic F127) micelles in water-swollen with various perfume oils.^44^

**Table 3:** Dimensions of the MEs Droplets Were Obtained by Fitting the SAXS Data into the Core-Shell Sphere Model^a^ ^b^ ^c^

Formulations	***r***_**c**_(nm)	***t**_**s**_* (nm)	***r* *_s_*** (nm)
ME	51.0	3.2	54.2
ME + menthol (0.1%m/m)	57.0	3.3	60.3
ME + menthol (1.0%m/m)	65.0	3.3	68.3

a *r*_*c*_, *t*_*s*_, and *r*_*s*_ represent the droplet’s core radius, shell thickness, and total radius, respectively.

b The estimated uncertainties for the fitted parameters are about ±10%.

c The PDI was assumed to be 0.10 ± 0.02 for all cases.

Although menthol does not significantly alter the structure of ME droplets, it is found to change the average size of the droplets. Based on the *r*_*c*_ values in Table 3, it can be suggested that its solubilization at 1.0% w/w increases the radius of the hydrophobic core of the original formulation by a factor of about 1.3, which is consistent with the DLS results (Table 2), where an increase in hydrodynamic diameter of about 1.2 times was observed in the Z-average values when comparing the unloaded ME with that containing 1.0% w/w of menthol. The *r*_*s*_ values for all tested ME also agree with the DLS results, with 2*r*_*s*_ ≈ Z-average.

Noticeable, the slight increase in turbidity observed in the formulation ME + menthol 1.0% was directly associated with the enlargement of droplet size resulting from menthol incorporation into the ME droplets, rather than the formation of crystalline structures (Supp Figure 1B). This finding supports that menthol was successfully encapsulated within the ME system, maintaining its physical stability and homogeneity.

Menthol possesses a hydroxyl group that imparts limited polarity due to the high electronegativity of oxygen; however, its solubility in water remains poor. This limited solubility arises from the predominance of hydrophobic moieties in its molecular structure, including the bulky cyclohexane ring and alkyl side chain, typical features of monoterpenes.^24^ Such structural elements reduce its affinity for aqueous environments, as reflected by its octanol/water partition coefficient (Log *P* ≈ 3.3), indicating a greater solubility in organic solvents and likely fatty acids.^45^

Additional evidence of menthol’s hydrophobic character derives from interfacial and wettability studies. For instance, eutectic menthol mixtures with octanoic acid have been reported to reduce surface tension and form biphasic systems with water at room temperature.^25,26^ Likewise, a cineole–menthol mixture (1:1) displayed a markedly reduced contact angle on glass (22.1°) compared with pure water (74.5°).^46,47^

The contact angle is a fundamental parameter of wettability, defined as the angle formed between a solid surface and the tangent to a liquid droplet at the point of contact.^46,47^ Its magnitude reflects the affinity between liquid and surface, such that low contact angles indicate strong spreading and high surface affinity, whereas high values denote poor wettability and a propensity for the liquid to bead. On surfaces with defined polarity, the contact angle is modulated by the polarity or hydrophobicity of the liquid. In this context, the low contact angle observed for menthol-containing mixtures on glass (22.1° vs 74.5° for water) highlights menthol’s enhanced spreading on nonpolar substrates. This behavior suggests that menthol preferentially distributes across hydrophobic domains rather than aqueous regions, favoring its incorporation into the oil-rich phase of ME.

Consistent with these reports, our SAXS analysis (Figure 2) did not show any indication of menthol crystals, which supports the hypothesis of menthol solubilization within the oil-rich domains of the ME droplets. This localization explains the modest increase in droplet size observed upon menthol addition while ruling out the formation of crystalline structures. Such preferential distribution within hydrophobic regions will likely influence droplet interfacial organization, stability, and drug release kinetics. These results demonstrate that menthol encapsulation in oil-rich phases is a key determinant of the formulated MEs’ structural arrangement and functional performance.

#### 3.1.1 Stability Studies of Fluid Menthol-Loaded ME

During the stability study, ME, ME + menthol 0.1% and ME + menthol 1.0% remained visually translucent, fluid, and homogeneous, with no evidence of phase separation, creaming, or changes in color or consistency throughout the 90-day evaluation period. The addition of menthol at a concentration of 1.0% led to an increase in formulation turbidity, as expected, which is consistent with an increase in the average droplet size. In addition, turbidity values tended to increase under refrigerated storage conditions, whereas only minor variations in pH and electrical conductivity were observed over time (Supp Figure 3).

pH stability is a critical parameter, as fluctuations may alter the physicochemical properties of the ME or the encapsulated compound, potentially affecting formulation stability, bioavailability, and biocompatibility, and ultimately compromising therapeutic safety and efficacy.^48^ The MEs exhibited a slightly acidic pH, ranging from 4.1 to 4.8 when stored at 30 °C and from 4.5 to 4.9 under refrigeration. Only minor pH variations were detected over the 90-day study period (Supp Figure 3A,B). Furthermore, the addition of menthol at concentrations of 0.1% (pH = 4.1–4.5 at 30 °C and 4.5–4.8 at 5 °C) or 1.0% (pH = 4.4–4.8 at 30 °C and 4.5–5.0 at 5 °C) did not induce abrupt or significant changes in pH values compared to those obtained on day 1, indicating good chemical stability.

Electrical conductivity measurements were used to characterize the type of ME based on the nature of the continuous phase. The relatively high conductivity values observed (49–88 μS/cm at 30 °C and 54–114 μS/cm at 5 °C) are indicative of an aqueous external phase^49,50^ (Supp Figure 3C,D). Importantly, conductivity values remained stable throughout the storage period, corroborating the maintenance of the microemulsion stability under both temperature conditions.

Regarding turbidity, the MEs initially exhibited overall low values (Supp Figure 3E,F). However, formulations containing 1.0% menthol consistently showed higher turbidity compared to menthol-free systems or those containing 0.1% menthol. Additionally, MEs stored at 5 °C exhibited higher turbidity than those stored at 30 °C. This temperature-dependent behavior suggests alterations in the supramolecular organization of the system under refrigerated conditions. At lower temperatures, reduced molecular mobility of surfactant and cosurfactant molecules may impair optimal interfacial packing, favoring an increase in mean droplet diameter and, consequently, higher turbidity values.

Since turbidity is directly proportional to particle size, these findings indicate that both low-temperature storage and higher menthol concentrations contribute to droplet size enlargement, likely due to changes in interfacial curvature and partial restructuring of ME domains. Despite these effects, all formulations remained physically stable over the 90 days, with no macroscopic signs of instability. A limitation of the present stability study is the absence of DLS measurements, which would enable direct monitoring of droplet size distribution and polydispersity over time. However, turbidity provides an indirect indication of changes in particle size; future studies combining long-term stability assays with DLS analysis will be essential to characterize nanoscale structural evolution further.

The thermodynamic stability of MEs is associated with their spontaneous formation and energetically favorable equilibrium state (Δ*G* < 0), in which the system remains stable over time without phase separation. This stability arises from the minimization of Gibbs free energy and fundamentally differs from kinetic stability, which depends on the presence of energy barriers and the time required for a system to reach equilibrium.^51^ However, the response of MEs to temperature variations involves changes in key thermodynamic parameters, including Gibbs free energy (ΔG), enthalpy (Δ*H*), and entropy (Δ*S*). Such changes can shift the equilibrium state of the system, affecting critical properties such as the critical micelle concentration, phase behavior, interfacial curvature, and overall dispersion stability. Although MEs are often described as thermodynamically stable systems, this stability is inherently restricted to a specific temperature range. Phase diagrams are typically constructed under fixed conditions and may not remain valid outside this defined interval.

Recent studies, such as that by Musakhanian (2024), highlight that even minor temperature variations can significantly alter the thermal phase domains of MEs, potentially inducing structural rearrangements or, in extreme cases, phase separation.^52^ Additionally, including those reported by Zhang et al., further demonstrates that temperature fluctuations near stability boundaries may trigger reversible transitions between different microemulsion types (e.g., from Winsor IV to Winsor II), followed by changes in droplet size and internal organization.^53^

In the present study, the increase in turbidity observed at lower storage temperatures, particularly in formulations containing higher menthol concentrations, suggests that subtle supramolecular rearrangements are occurring rather than a loss of stability. These findings support the concept that thermodynamic stability ensures the formation and persistence of microemulsions only within a defined set of conditions, while temperature plays a decisive role in delimiting the boundaries of this stability window. Outside the optimal range, even energetically favored systems may undergo structural reorganization, phase transitions, or functional alterations, without necessarily exhibiting macroscopic instability.

### 3.2 Characterization of men-XG-ME Formulations

Once we understood the effects of menthol alone, we sought to understand the influence of menthol and XG in a formulation. We selected concentrations close to 0.1% due to menthol’s ability to induce a cooling sensation in the skin.^54,55^

TEM was employed to visualize the supramolecular structures of XG-thickened microemulsions (men-XG-ME1 to ME5). In samples with lower XG concentrations (men-XG-ME1 and ME2), only limited formation of the polymeric network was observed. By contrast, formulations with higher XG content (men-XG-ME3, ME4, and ME5) exhibited a more pronounced polymeric mesh, even after dilution, with ME droplets visibly associated with the polymer network. This morphology is characteristic of XG gels and suggests that ME droplets are physically integrated into the XG matrix through intermolecular interactions, such as hydrogen bonding.^56,57^ Formulations men-XG-ME6 and ME7 correspond to the central point in the DOE and contain the same concentrations of menthol and XG as men-XG-ME5, exhibiting similar structural features.

Noteworthy, the droplets in the presence of XG appeared less spherical than those observed without polymer (Figures 1 and 3). This morphological difference may arise from interactions between droplets and the XG network and from inherent limitations of TEM sample preparation, particularly dehydration artifacts. Despite these changes, ME droplets coexisted with the XG network without substantially disrupting its structural organization.^58,59^ This network arrangement was further supported by SAXS data (Figure 4), which is discussed in detail later in this section.

**Figure 3:**
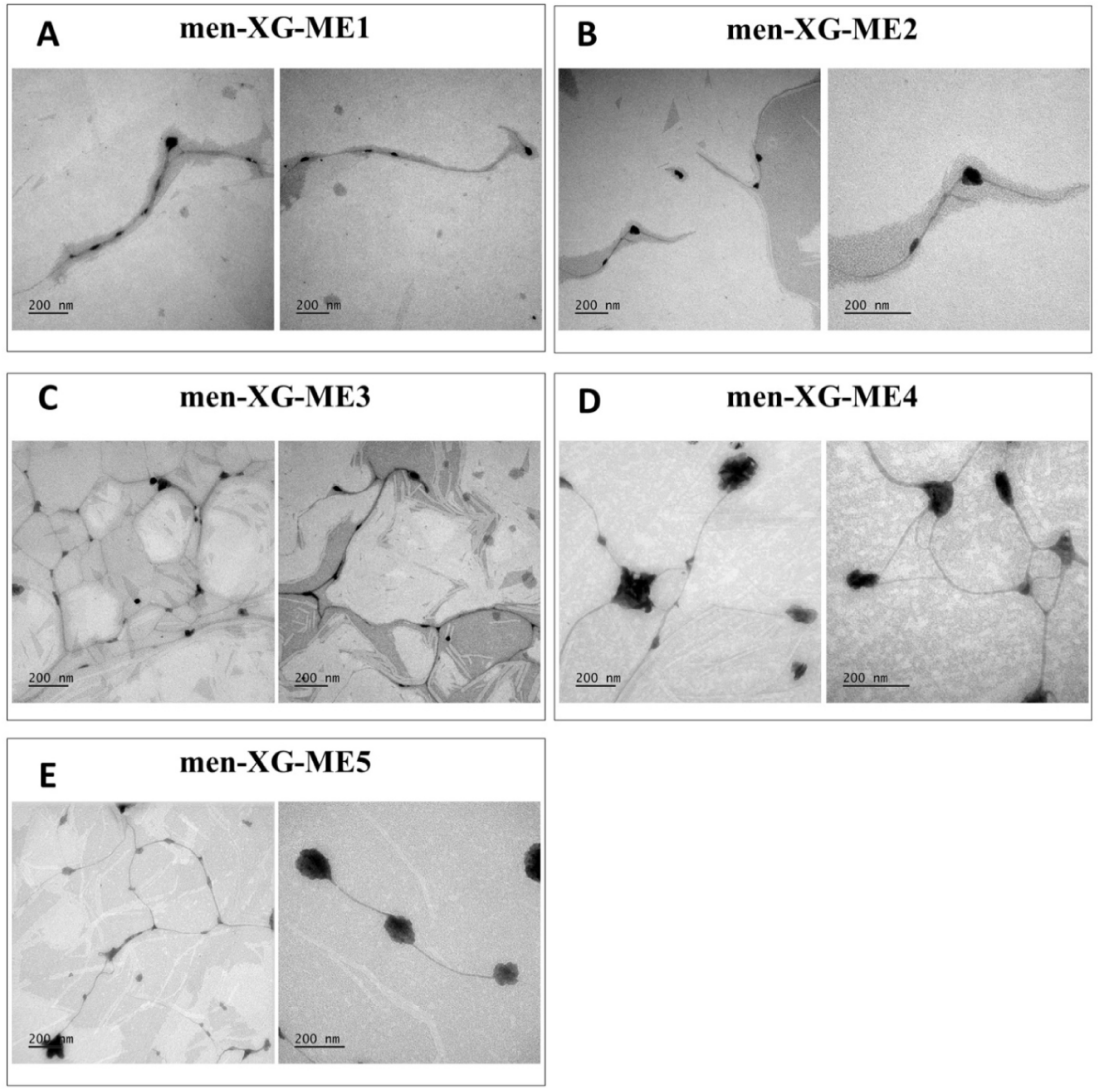
TEM images of menthol-loaded xanthan gum-modified microemulsions (men-XG-ME1 to ME5). (A) men-XG-ME1, (B) men-XG-ME2, (C) men-XG-ME3, (D) men-XG-ME4, and (E) men-XG-ME5. Scale bar: 200 nm.

**Figure 4:**
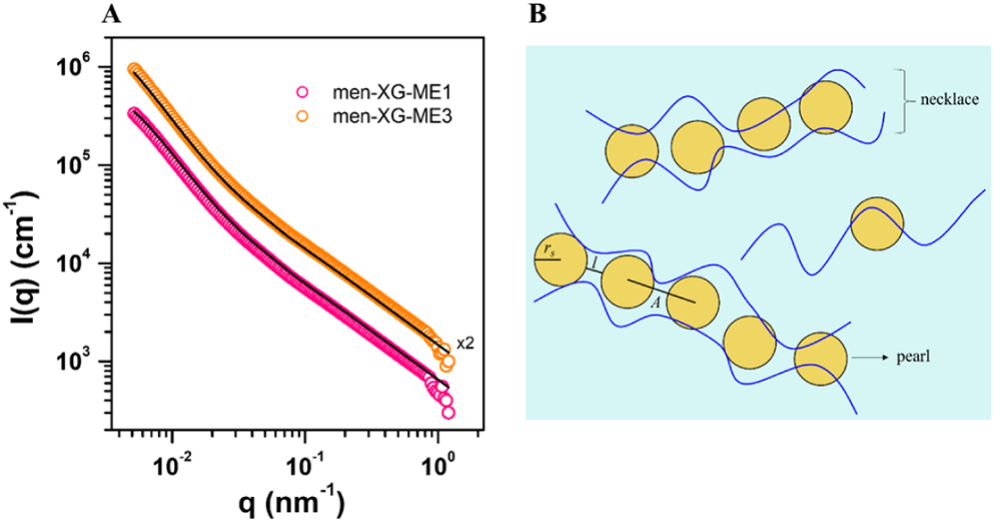
(A) SAXS curves (open dots) for the two analyzed MEs containing XG and the fits (black lines) to the pearl-necklace model. The data are shifted along the *y*-axis for better visualization. (B) Schematic representation (not to scale) of the pearl-necklace model applied to the samples with the corresponding dimensions resulting from the fits. ME droplets are yellow spheres, while XG chains are blue, slightly curved lines. The dimensions *r*_s_, *A*, and *l* represent the mean radius of the chained spheres (droplets), the edge distance, and the thickness of the chain connection, respectively.

Furthermore, the incorporation of menthol and XG did not markedly alter droplet morphology, preserving the overall microstructural organization of the system. These findings confirm the successful formation of well-defined microemulsions within XG-thickened gel formulations.

XG is well-known for its ability to establish hydrogen bonds with various formulation components, including Tween 80 and the propylene glycol.^56,57^ These hydrogen bonding interactions are critical for enhancing the ME stability by promoting forming a thicker interfacial film around the droplets, thereby reducing the likelihood of coalescence. Additionally, by tailoring the hydrophilicity of the system, propylene glycol may further facilitate the formation and persistence of hydrogen bonds, contributing to the stabilization of the microemulsion. Beyond hydrogen bonding, it is also recognized that other noncovalent interactions, such as van der Waals forces, may act synergistically within the system, influencing its internal organization and stability.^60^

From a rheological perspective, hydrogen bonding between XG, Tween 80, and propylene glycol molecules increases viscosity and induces shear-thinning behavior, as commonly seen in non-Newtonian fluids.^56,61^ The results presented in Table 4 indicate that the average droplet (Z-Ave and Peak 1) size in the DOE samples did not exhibit statistically significant differences. This finding suggests that incorporating different XG concentrations (0.1%m/m, 0.3%m/m, and 0.5%m/m) maintained uniformity in the average droplet size of the MEs, regardless of the changes in the composition. In addition, the observed increase in PDI might be due to the presence of free and aggregated XG chains after sample dilution and not by the modification on droplet size as indicated by the distribution graphics (Supp Figure 4) and the Peak 1 values (Table 4), where it is possible to see a maintained of the structure around 102–111 nm in all samples.

**Table 4:** Z-Average (nm) and Polydispersity Index (PDI) Determined by DLS, from men-XG-ME1 to 7^a^ ^,^ ^b^

Formulations	Z-Ave (nm)	Peak 1 (nm)	PDI
men-XG-ME1	79 ± 2	107 ± 5	0.26 ± 0.00
men-XG-ME2	81 ± 2	111 ± 3	0.24 ± 0.01
men-XG-ME3	79 ± 2	110 ± 5	0.28 ± 0.02*
men-XG-ME4	84 ± 2	108 ± 1	0.23 ± 0.01^##^
men-XG-ME5	82 ± 3	102 ± 6	0.25 ± 0.00
men-XG-ME6	80 ± 3	108 ± 9	0.24 ± 0.01^#^
men-XG-ME7	79 ± 1	108 ± 6	0.24 ± 0.01^#^

a Data are given as average ± SD (*N* = 3).

b One-way ANOVA followed by Tukey posttest. (PDI: *p < 0.05 vs men-XG-ME2; #*p* < 0.05, ##*p* < 0.01 vs men-XG-ME3). Z-Ave.: no significant difference between samples.

Nevertheless, these results suggest that the droplets coexist stably with the XG network without significantly altering the properties of the ME, as previously indicated by TEM and later by SAXS.

The relationship between viscosity and the hydrodynamic diameter (DH) of droplets is also relevant for interpreting the results. According to the Stokes–Einstein equation, the DH of particles in thermal equilibrium with the solvent is inversely proportional to the medium’s viscosity (η).^62^ Notably, no differences in droplet size (Z-Ave and Peak 1) were observed among the formulations. Variations in viscosity and DH can significantly impact pharmaceutical and cosmetic performance, with increased viscosity enhancing formulation stability by reducing coalescence and sedimentation while improving topical retention and adhesion for cutaneous applications.^63^ Consequently, XG incorporation not only tailors physical and chemical properties but may also positively influence the controlled release and efficacy of the incorporated active compound, such as menthol. Thus, it is essential to emphasize that the optimal XG concentration must balance system stability with formulation performance. While higher concentrations can increase viscosity (Figure 5, Supp Table 1) and promote greater stability, excessively high concentrations may result in undesirable effects, such as handling difficulties and negative impacts on sensorial aspects of the formulation.

**Figure 5:**
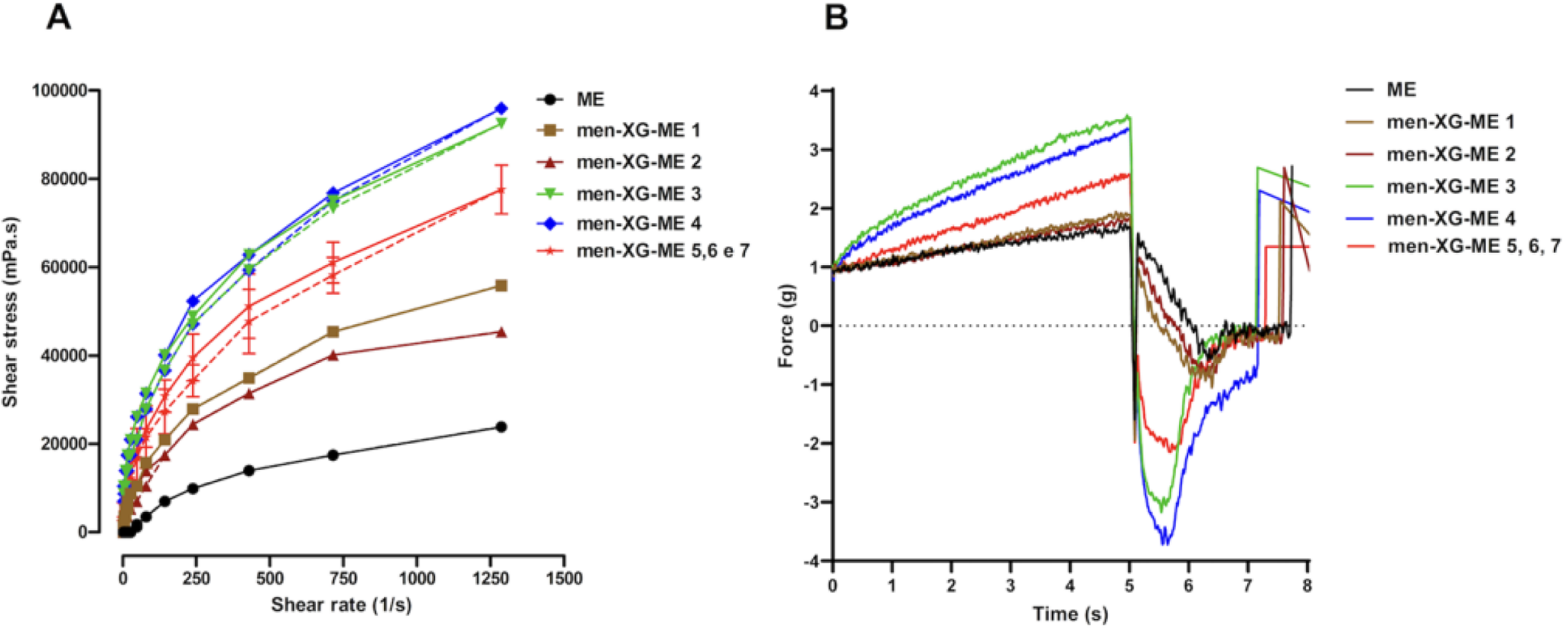
Rheological and textural characterization of all men-XG-ME formulation in the factorial design. (A) Rheogram. (B) Texture Profile.

SAXS was used to analyze all samples and further investigate the effect of XG on the supramolecular structure of the men-XG-ME (1 to 7) formulations. Figure 4A shows the SAXS curves obtained for men-XG-ME formulations with the same amount of menthol and two increasing concentrations of XG (Table 1). These data show an apparent change in the shape factors compared to MEs without added XG (Figure 2A). According to the fits (black lines in Figure 4A), the addition of XG led to the formation of polysaccharide chains linked to the ME droplets, forming cylindrical-like nanostructures often referred to as a “pearl-necklace” (Figure 4B), which is consistent with the structures seen in the TEM images presented in Figure 3. Similar results were found for other investigated compositions (Supp Figure 5). According to the literature,^64^ such polyelectrolyte-MEs complexes can have an elongated or compact shape, which depends on various parameters, especially on the stiffness of the polyelectrolyte chain.

As has been reported for other anionic biopolymers, including sodium carboxymethylcellulose and sodium hyaluronate,^65^ the high rigidity of ionic polysaccharide chains such as XG promotes the formation of cylindrical structures with relatively low flexibility when mixed with MEs. Although it is reported^64–66^ that the formation of such systems is mainly driven by the electrostatic interactions between oppositely charged polysaccharides and the surfactants that compose the MEs, the occurrence of such ordered systems has also been found for MEs consisting of nonionic surfactants,^67,68^ as in the current work.

It is known that the formation of these cylinder-like structures has a significant impact on the rheological properties of the resulting formulations. For example, this has been reported previously for gels of sodium hyaluronate and cationic surfactants^69^ and for aqueous mixtures of cellulose-based polycation (JR 400) and anionic surfactants.^70^ In the first case, the authors found that the viscoelastic properties of the gel can be fine-tuned by the polymer’s molecular weight and the surfactant’s structure. In contrast, in the second case, it was found that blends with an excess of polycation form a highly viscous network structure due to cylindrical aggregates of surfactant and polyelectrolytes linked together by the long JR 400 chains.

Therefore, the increase in the viscosity of the formulations observed in our study can be correlated with the appearance of such structures when the concentration of XG is increased. For example, suppose the cylinder-like structures are long and densely packed, as assumed in the present work. In that case, the structure formed can hinder the flow during shear, leading to increased shear stress, as shown in Figure 5A. The cylinder-like structure also leads to a higher viscosity, which can affect other essential pharmaceutical application properties, such as consistency and texture, as detailed later.

From the fitted data in Figure 4A, the mean radius of the chained spheres (*r*_*s*_), the edge distance (*A*), and the thickness of the chain linkage (*l*) can be estimated, as illustrated in Figure 4B. The mean number of pearls (*n*) per cylinder can also be calculated. The determined dimensional parameters are listed in Table 5. The results are approximately insensitive to the XG concentration, which increased from 0.1% to 0.5% for the two MEs studied. The *r*_*s*_ values are consistent with those derived from DLS (Tables 2 and 4) and SAXS (Table 3) for XG-free emulsions, indicating that the complex structures are formed such that the ME droplets retain their original structure. The other two parameters (*A* and *l*) are also consistent, considering *A* ≈ 2*r*_*s*_ + *l* (see Figure 4B).

**Table 5:** Dimensions of the ME with Two Increasing Amounts of XG Obtained by Fitting the SAXS Data to the Pearl-Necklace Model^a^ ^,^ ^b^ ^,^ ^c^ ^,^ ^d^

Formulations	***r** **_s_*** (nm)	***A*** (nm)	***l*** (nm)	*n*
men-XG-ME 1	51.0	121.0	20.0	25
men-XG-ME 3	51.0	123.0	22.0	27

a *r_s_*, *A*, and *L* stand for the mean radius of the chained spheres (drops), the edge distance and the thickness of the chain linkage, Respectively.

b *N* stands for the mean number of pearls per cylinder.

c The estimated uncertainties for the fitted parameters are approximately ±10%.

d The PDI was assumed to be 0.10 ± 0.02 for all Cases.

The number of pearls in the necklace (*n*) is quite large compared to other systems reported in the literature with synthetic polyelectrolytes.^65^ It could be a consequence of the relatively large contour length of a fully extended XG chain. The literature^71^ states that XG can have contour lengths of up to 5000 nm due to its high rigidity and molecular weight, making it capable of forming complexes with considerable amounts of ME droplets.

### 3.3 Rheological Profile and Texture Analysis of men-XG-MEs

The rheological behavior of the men-XG-ME formulations was evaluated using the Ostwald model. This model has two key parameters: the consistency index (*k*), which reflects a formulation’s resistance to flow, and the flow index (*n*), which indicates the extent of pseudoplastic (shear-thinning) behavior.

As shown in Figure 5 and Supporting Information Table S1, the addition of XG significantly increased the consistency index, confirming that XG enhances the viscosity of the formulations. Increasing viscosity is desirable in topical delivery systems, as it improves formulation retention on the skin. All formulations exhibited a flow index below 1 (Supporting Information Table S1), expected in pseudoplastic behavior, in which viscosity decreases with increasing shear rate. Adding XG further enhanced the shear-thinning profile (Supporting Information Tables S1, S9 and S10). The literature typically describes this behavior for aqueous dispersions and gels containing XG.^17,18^ Such rheological features are particularly advantageous for dermatologic formulations, facilitating their clinical use.

Interestingly, the changes in flow behavior observed during rheological analysis were not initially predicted by SAXS, which highlights the complementary nature of these analytical techniques in characterizing supramolecular structures.

At the same time, texture profile analysis (TPA) was performed to investigate the mechanical properties of the formulations, such as firmness, consistency, adhesiveness, and cohesiveness (see Figure 5B and Supporting Table S1). A concentration-dependent trend was noted, where higher concentrations of XG (men-XG-ME 3 to 7) significantly increased all textural parameters. In contrast, formulations with lower XG content (men-XG-ME 1 and 2) exhibited texture profiles similar to those of the unmodified liquid ME.

Firmness is the maximum force required to deform the sample. In our study, the rise of XG concentration led to its increase (Supp Figures 6 and 7). This phenomenon is likely due to establishing an intricate XG polymeric network. Analysis of variance confirmed that XG was the primary factor influencing firmness (*p* < 0.05). Similar results have been previously reported, with XG enhancing the firmness and cohesiveness of gel formulations.^72,73^ For example, Alves et al. (2020) showed that increased XG content led to stiffer gels, with minor variations occurring when xanthan/konjac glucomannan ratios were adjusted.^72^

Consistency, related to formulation viscosity, also increased with XG content. This parameter was assessed through both TPA and rheological measurements, which showed good agreement. A significant quadratic effect of XG concentration on consistency was observed (*p* < 0.001), reinforcing the role of XG in modulating formulation thickness and flow resistance. These results are consistent with findings by Djekic et al. (2016), who reported that higher XG concentrations led to increased viscosity, consistency, and mucoadhesiveness in MEs loaded with econazole nitrate.^17,74^

Adhesiveness, representing the work required to remove the sample from a surface, was influenced by both XG and menthol. This parameter, measured as the area under the negative curve in the TPA, is relevant to the formulation’s dermatological use. XG contributed to increased adhesiveness through its ability to form cohesive and sticky networks—an attribute frequently exploited in pharmaceutical gels.^63,74^ Concomitantly, menthol, being hydrophobic and oil-soluble, likely contributed to increased droplet size and altered interfacial properties, possibly enhancing adhesion by expanding the organic phase volume, as observed in oil-rich emulsions.^75^

Cohesiveness, which measures the internal structural integrity of the formulation, was primarily influenced by XG. Higher cohesiveness values indicate stronger intermolecular forces, as in intricate polymeric networks, and may affect the formulation’s ability to maintain its structure upon application.^76^

Cohesiveness, a measure of the internal structural integrity of the formulation, was primarily governed by XG. Higher cohesiveness values reflect stronger intermolecular forces and may influence the formulation’s ability to maintain structure upon application.^76^

In summary, the addition of XG significantly altered the rheological and textural characteristics of the men-XG-ME formulations. Higher concentrations of XG were associated with increases in viscosity, firmness, consistency, adhesiveness, and cohesiveness. These findings suggest that XG could be an effective excipient for enhancing the mechanical properties of microemulsion-based topical systems, similar to well-known aqueous solutions.^11^

#### 3.3.1 Rheology and TPA Correlation

After a thorough physical characterization of the men-XG-ME formulations and data modeling (Supporting Information), evaluating the correlation between texture and rheological properties is essential. This correlation plays a critical role in the product development processes of the cosmetics and pharmaceutical industries.^77–80^

By exploring the relationship between sensory attributes, such as texture, and mechanical properties, like rheology, we expect to be able to customize formulation properties to meet specific needs. Rheological analysis evaluates properties such as viscosity and elasticity by applying mechanical forces or shear rates. On the other hand, texture analysis aims to replicate human sensory perception.^7,78,81^ The differences in these approaches are related to the distinct nature of each mechanical analysis. Thus, it is possible for a product to exhibit high viscosity in rheological measurements but still feel soft during its use. Understanding these differences is crucial, especially when correlating physical and sensory properties to optimize product formulation and meet consumer expectations.^7,78,81^

Adjusting ingredient concentrations to achieve desired properties by correlating texture and rheological parameters is possible. This optimization approach, guided by quality-by-design principles, can improve consumer experience, foster formulation innovation, streamline production, and provide a solid scientific foundation for decision-making.^80^

Table S1 (Supporting Information) presents the measured physical properties, demonstrating high reproducibility in sample preparation. The rheological and texture profile analysis (TPA) results varied significantly in response to changes in processing conditions. Pearson’s correlation analysis (Table 6) revealed strong correlations between the consistency index and firmness (*r* = 0.9751) as well as cohesiveness (*r* = −0.9028). In contrast, the correlations with consistency and adhesiveness were moderate (*r* = 0.8657 and *r* = −0.8813, respectively). The flow index exhibited moderate correlations with firmness, cohesiveness, and adhesiveness, though it showed a weak correlation with consistency.

**Table 6:** Pearson’s Correlation Coefficients between the Analysis Responses of XG (X1) and Menthol (X2)^a^

	Firmness (g)	Consistency (g·s)	Adhesiveness (g·s)	Cohesiveness (g)
Consistency index (Pa·s^*n*^)	0.9751***	0.8657*	–0.8813**	–0.9028**
Flow index	–0,8689*	–0,7144	0,8314*	0,7783*

a (***p < 0.001, **p < 0.01, *p < 0.05).

Gilberto et al. (2013) demonstrated the positive influence of polymers on viscosity and viscoelastic parameters, along with significant correlations between rheology and texture analysis.^77^ Calixto et al. (2018) also reported strong correlations between consistency index and texture analysis (*R* ^2^ = 0.869 to 0.981), while correlations between flow index and texture analysis were weak (*R* ^2^ = −0.010 to 0.102).^78^

To investigate the associations between rheological parameters (consistency index *k* and flow index *n*) and TPA parameters (firmness, consistency, adhesiveness, and cohesiveness), five regression models were tested: linear, polynomial, logarithmic, power, and exponential (Supporting Information Table S11). Figure 6 presents representative comparisons between the two best-fitting models, linear and polynomial, while the complete set of equations and determination coefficients (*R*²) are provided in Supporting Information Table S11.

**Figure 6:**
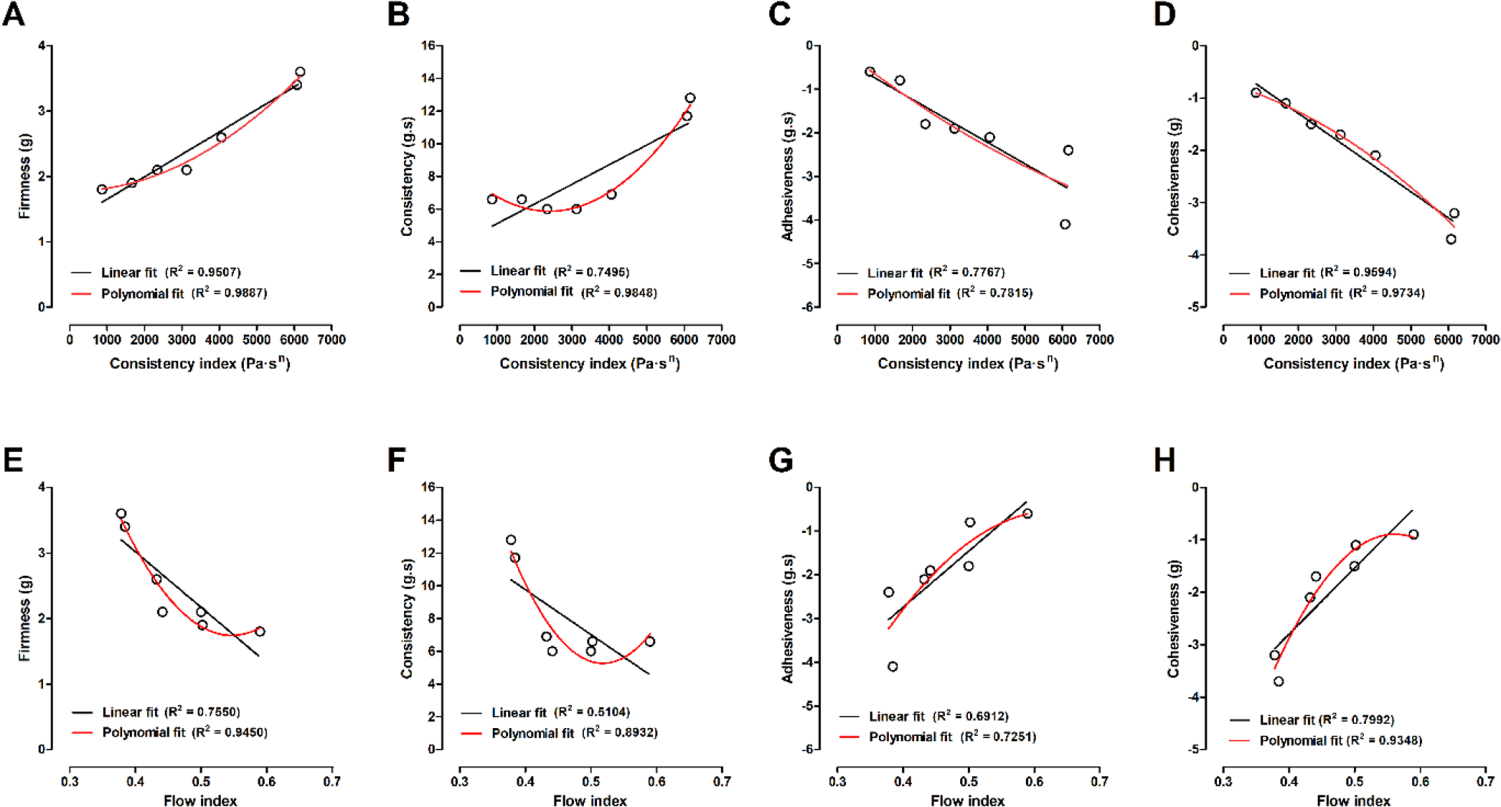
Linear plots between rheological and TPA measurements. The interaction between the (A) consistency index (Pa·s^*n*^) and firmness (g), (B). consistency (g·s), (C) adhesiveness (g·s), (D) cohesiveness (g). The interaction between (E) the flow index and firmness (g), (F) consistency (g·s), (G) adhesiveness (g·s), and (H) cohesiveness (g) Linear fit (black lines); Polynomial fit (red lines).

Polynomial models occasionally achieved higher *R*² values compared with linear regressions. For instance, improvements were observed for *k* vs firmness (0.9887 vs 0.9507), *k* vs consistency (0.9848 vs 0.7495), *k* vs cohesiveness (0.9734 vs 0.9594), and to a lesser extent, *k* vs adhesiveness (0.7815 vs 0.7767). Similarly, for *n*, polynomial regression outperformed linear fits in *n* vs firmness (0.9450 vs 0.7550) and *n* vs cohesiveness (0.9348 vs 0.7992).

These trends are consistent with the quadratic curvature identified in the response surface methodology (RSM) analyses (Supporting Information Tables S2, S5, and S10). The significance of curvature at the central points of the experimental design suggests that the responses are not strictly linear but instead exhibit an optimized effect at intermediate factor levels (X1 and X12). This observation supports the likelihood of using polynomial adjustments in certain situations, as these models can effectively capture the nonlinear behaviors identified through RSM analyses. These findings suggest that polynomial models may better represent subtle curvilinear trends and optimized responses. In contrast, linear models are often more appropriate for broader mechanistic interpretations.

Nevertheless, it is well established in the literature that polynomial regression is prone to overfitting and may produce unrealistic oscillations or artifacts (commonly referred to as Runge’s phenomenon), especially at higher polynomial orders.^82,83^ While polynomial models provide mathematical flexibility, they often perform poorly outside the calibration range and offer limited physical interpretability. In contrast, linear regressions offer clear mechanistic insights, require fewer parameters, and minimize the risk of modeling noise instead of genuine structure–property relationships. This approach aligns with best practices in regression modeling.^84^

The linear models offer a straightforward and physically interpretable framework for understanding how interactions between droplets and the xanthan gum network affect texture and flow behavior. These simpler linear regression models are less likely to overfit the data, making them preferable to more complex nonlinear alternatives. This approach ensures that the presented relationships are both statistically robust and mechanistically plausible, minimizing the risk of introducing misleading artifacts.

## 4 Conclusion

This comprehensive multiscale investigation successfully revealed the intricate physicochemical and supramolecular characteristics of oleic acid-based microemulsions modified with XG and incorporating menthol, achieving our primary objective of establishing quantitative structure–property correlations through advanced analytical approaches.

Our findings showed that menthol incorporation (0.1–1.0% w/w) produces concentration-dependent droplet size modulation while preserving the fundamental supramolecular architecture of the microemulsion system. The complementary SAXS, TEM, and DLS analyses provided evidence that menthol preferentially partitions within the hydrophobic oil droplets, increasing core radius by a factor of 1.3-fold at 1.0% loading without disrupting the spherical morphology or interfacial organization. This molecular level provides insights for optimizing drug loading in similar colloidal systems.

The addition of XG revealed a structural complexity, with SAXS analysis unveiling a “pearl-necklace” supramolecular architecture that transforms the system’s nanoscale organization. This discovery is an advance in the understanding of biopolymer-microemulsion interactions and demonstrates how polysaccharide networks can create hierarchical structures that bridge molecular and macroscopic scales. It is noteworthy that our systematic approach revealed that light-based techniques were insufficient to detect the subtle but critical concentration-dependent variations, emphasizing the necessity of mechanical testing approaches—specifically rheological profiling and texture analysis—to fully capture the functional implications of these structural modifications.

The rheological and textural characterization established clear correlations between nanoscale organization and macroscopic performance, with XG concentration emerging as the dominant factor controlling flow behavior and mechanical properties, while menthol exhibited more subtle effects, such as adhesiveness.

This work presents a framework for characterizing biopolymer-modified microemulsions by connecting molecular interactions to their macroscopic properties, thereby addressing existing gaps in colloidal science. By utilizing a multiscale approach, this study offers a comprehensive methodology for creating more effective formulations for dermatological applications.

## Supplementary Material



## Data Availability

The data supporting this study are available within the manuscript and Supporting Information file.

## Notes

Pseudoternary phase diagrams; TEM images of ME and ME + menthol droplets; DLS size distribution reports for ME and men-XG-ME1 to 7; SAXS fitting and SAXS curves for ME containing XG; physicochemical characterization of ME with xanthan gum (X1) and menthol (X2); regression analysis for variables X1 and X2; detailed statistical data from rheology and texture analyses (PDF)

## Abbreviations

ME: Microemulsion
XG: Xanthan gum
men: Menthol
men-XG-ME: Menthol-xanthan gum-loaded ME
SAXS: Small-angle X-ray scattering
TEM: Transmission electron microscopy
TPA: Texture profile analysis
DLS: Dynamic light scattering
Z-Ave: Z-average
PDI: Polydispersity Index
