# Supplementary material for: Unveiling Supramolecular Structures Formed by Menthol and Xanthan Gum in Oleic Acid-Based Microemulsions
Source: ACS Omega. 2026 Jan 29;11(5):8117–30. doi: 10.1021/acsomega.5c10561 (PMC12902958; doi:10.1021/acsomega.5c10561)
Supplement: Supplementary file 1 [file ao5c10561_si_001.pdf]

SUPPORTING INFORMATION

UNVEILING SUPRAMOLECULAR STRUCTURES FORMED BY MENTHOL AND XANTHAN GUM IN OLEIC ACID-BASED MICROEMULSIONS

Rafael Leonne Cruz de Jesus[a], Letícia Maria Silva Amaral[b], Tainá Santos Souza[a], Guilherme A. Ferreira[c], Bruna Aparecida Souza Machado[d], Diogo Rodrigo Magalhães Moreira[e], Henrique Rodrigues Marcelino[a,b,*], and Darizy Flávia Silva [a,f,]

[a] Graduation Program in Pharmacy, College of Pharmacy, Federal University of Bahia, Salvador, Bahia, Brazil, 40170-115
[b] Department of Medicines, College of Pharmacy, Federal University of Bahia, Salvador, Bahia, Brazil, 40170-115
[c] Department of Physical Chemistry, Institute of Chemistry, Federal University of Bahia, Salvador, Bahia, Brazil, 40170-115
[d] SENAI Institute of Innovation (ISI) in Health Advanced Systems (CIMATEC ISI SAS), University Center SENAI/CIMATEC, Salvador, 41650-010, Brazil
[e] Fundação Oswaldo Cruz, Instituto Gonçalo Moniz, CEP 40296-710, Salvador, BA, Brazil.
[f] Department of Bioregulation, Institute Health Sciences, Federal University of Bahia, Salvador, Bahia, Brazil, 41100-110

*Corresponding author:
Prof. Henrique Rodrigues Marcelino, Ph.D.
Department of Medicines, College of Pharmacy, Federal University of Bahia,
Rua Barão de Jeremoabo, 147, Ondina, Salvador/BA, 40170-115, Brazil.
Email: henrique.marcelino@ufba.br

Pseudo-ternary Phase Diagram

The pseudoternary phase diagram was constructed to identify the concentration ranges yielding a Winsor IV-type, characterized by a translucent, fluid, and homogeneous appearance blue region. Other regions included highly viscous translucent systems (black), turbid conventional emulsions (green), and turbid viscous systems (purple). These observations align with previous reports involving triptolide-based microemulsions. The formulation selected for further investigation was chosen based on its favorable physical characteristics (Supp figure 1A).

Comparable macroscopic properties were observed in formulations with different menthol concentrations (0.1% w/w and 1.0% w/w), although the sample containing 1.0% menthol exhibited a slight increase in turbidity (Supp figure 1B). This phenomenon was further investigated using dynamic light scattering (DLS) and small-angle X-ray scattering (SAXS). The results demonstrated that the increase in turbidity was associated with a larger droplet size caused by menthol incorporation into the microemulsion droplets, rather than the formation of crystalline structures. These findings support the hypothesis that menthol was successfully encapsulated within the microemulsion system.

Figure S1 A. Pseudoternary phase diagram of the oil-surfactant-water system constructed at 25 °C using a surfactant/cosurfactant mixture composed of Tween 80 and propylene glycol at a 2:1 (w/w) ratio. The blue region corresponds to translucent, fluid, and homogeneous formulations, characteristic of a single-phase region (Winsor IV). The remaining regions are defined as follows: red - phase separation; black - translucent, liquid, and homogeneous systems; green - opaque, liquid, and homogeneous systems; purple - opaque, viscous, and homogeneous systems. **B.** Turbidity of Microemulsion (ME); ME + menthol (0.1% w/w), ME + menthol (1.0% w/w). One-way ANOVA followed by Tukey post-test. (*p<0.05 vs ME).

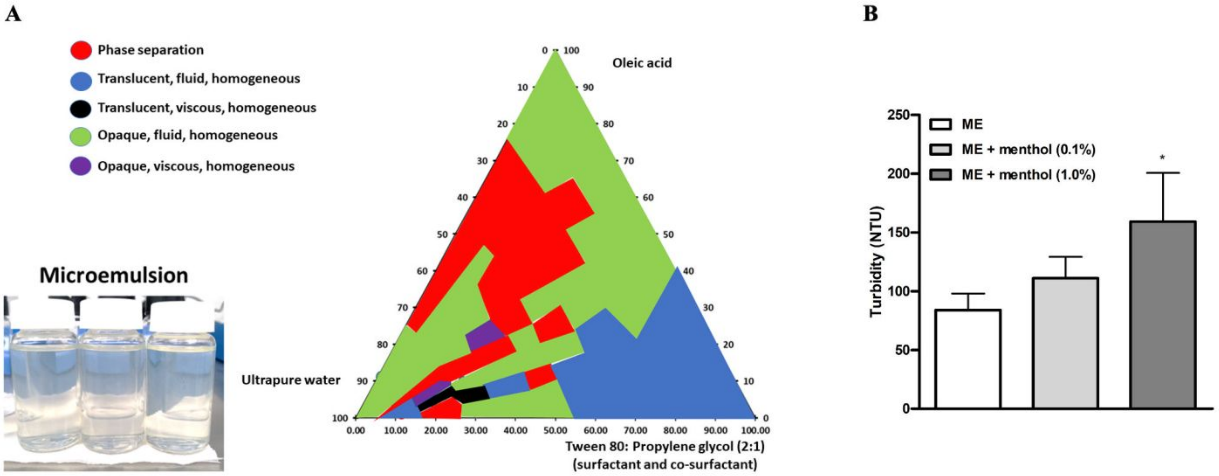

Transmission electron microscopy

Supplementary Figure 2 presents representative TEM images of droplets from the ME, ME + menthol (0.1% w/w), and ME + menthol (1.0% w/w) formulations prepared with 2.0% phosphotungstic acid (PTA) and subjected to 48 h of drying. While the micrographs predominantly show spherical droplets, some elongated or irregular morphologies were also observed. These deviations are likely artifacts introduced during the prolonged drying process required for TEM sample preparation, rather than reflecting intrinsic properties of the microemulsions. Overall, the images confirm the presence of discrete droplets dispersed within a continuous phase, consistent with the structural characteristics of microemulsion systems.

Figure S2. TEM images. **(A)** droplets from ME; **(B)** droplets from ME + menthol (0.1%w/w); **(C)** droplets from ME + menthol (1.0%w/w).

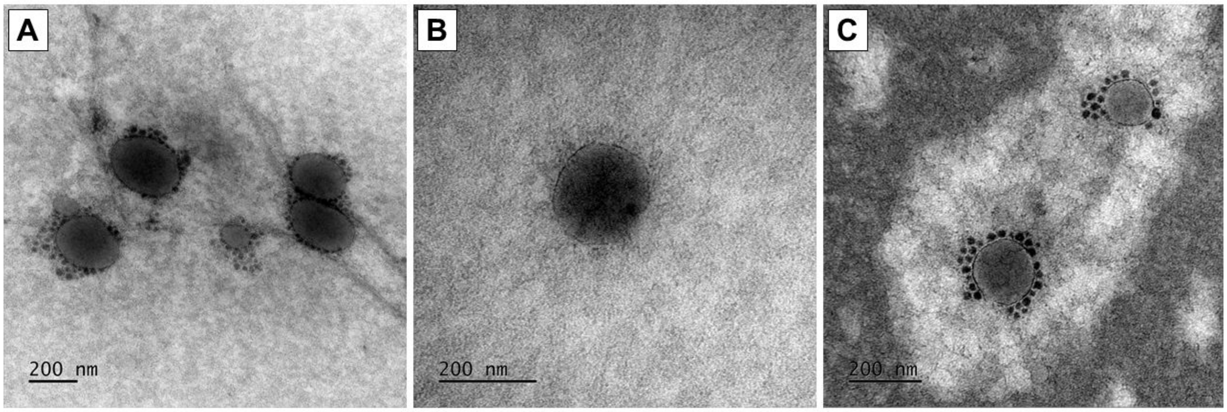

Figure S3. Stability study of menthol-loaded (0.1 or 1.0% w/w) and unloaded microemulsions over 90 days under different storage temperatures (5 or 30 °C). pH values (A and B), electrical conductivity (C and D), and turbidity (E and F). Data were analyzed using two-way ANOVA followed by Tukey's post hoc test. *p < 0.05 and **p < 0.01 vs. ME; #p < 0.05, ##p < 0.01, and ###p < 0.001 vs. menthol-loaded ME (0.1%); +p < 0.05 and ++p < 0.01 vs. menthol-loaded ME (1.0%). Statistical comparisons were performed relative to day 1 of the respective groups.

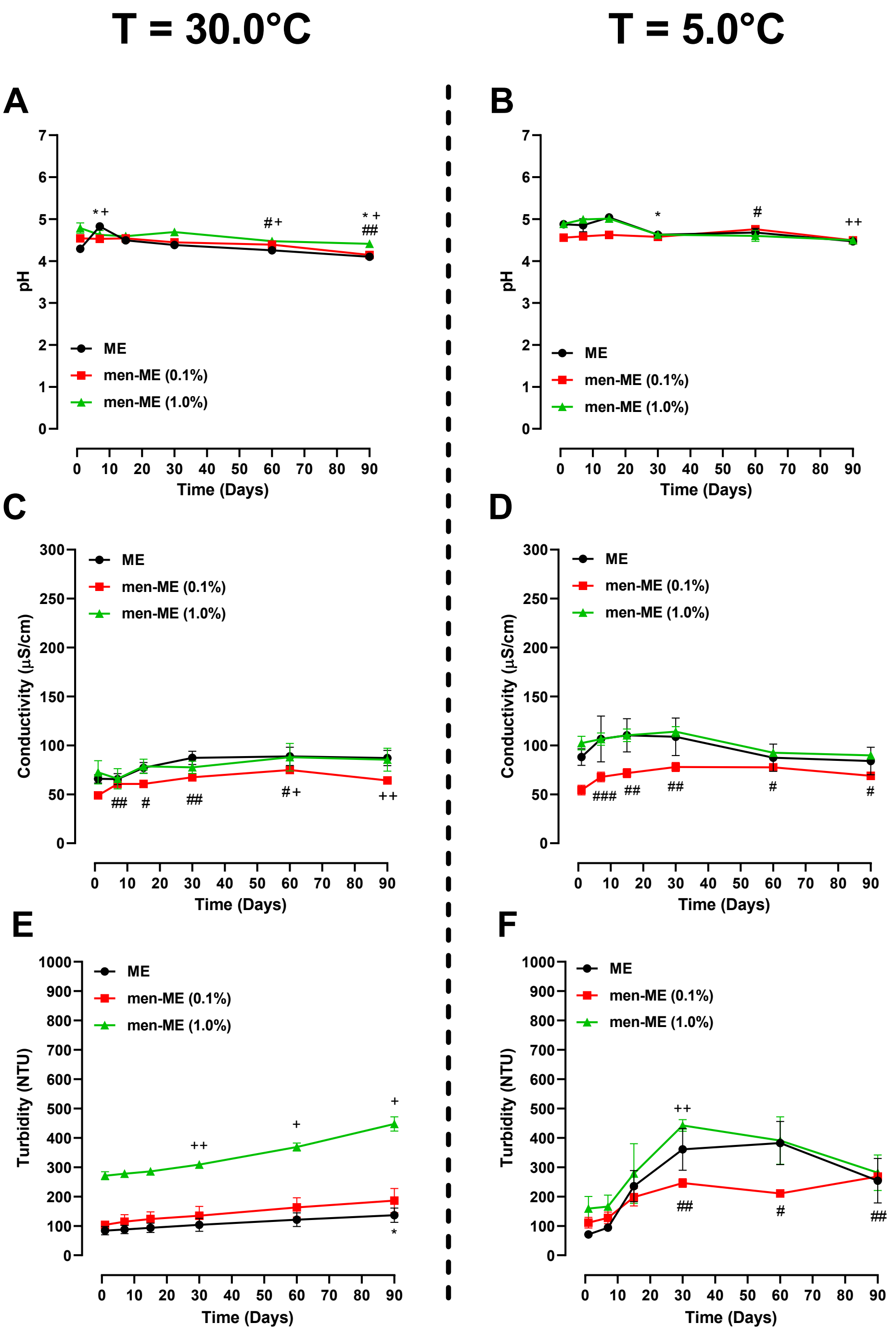

Dynamic Light Scattering

Supplementary Figure 4 presents the size distribution profiles by intensity obtained through DLS analysis for ME and men-XG-ME1 to ME7 formulations. The data provide a detailed overview of droplet size distribution, facilitating a clearer interpretation of the structural characteristics of each formulation.

Figure S4. DLS analysis of ME or men-XG-ME1 to 7, presenting the size distribution report by intensity.

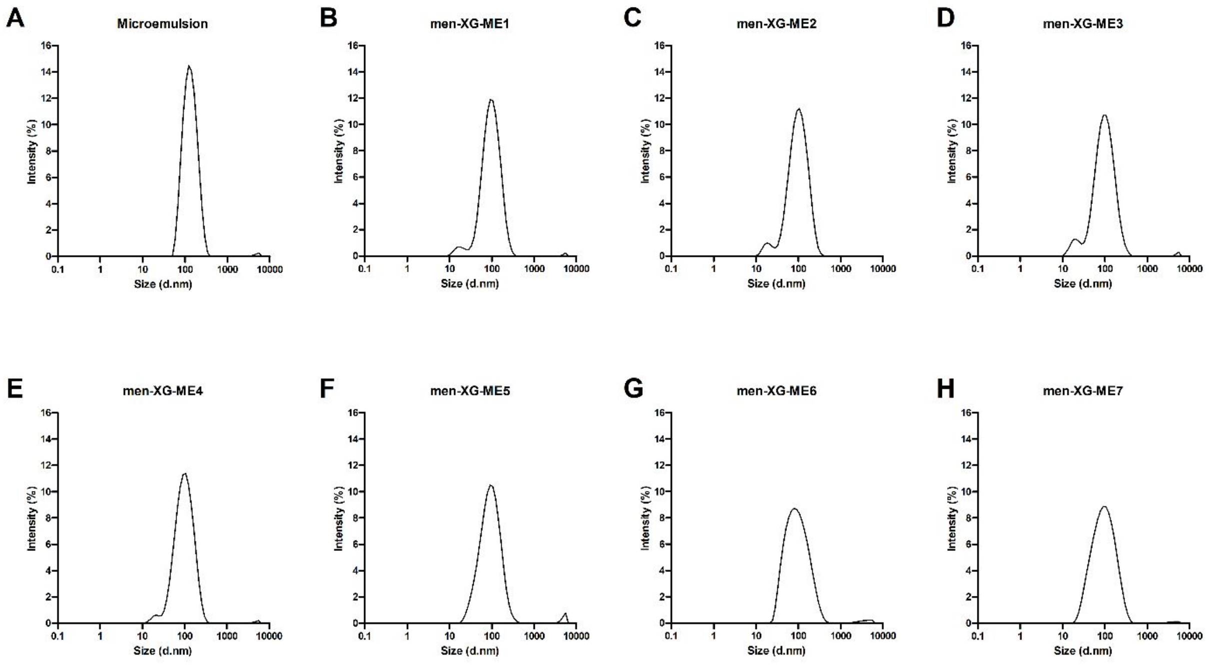

SAXS fitting

Small Angle X-ray Scattering (SAXS) profiles were analyzed using SasView (version 6.0.1, available at www.sasview.org), an open-source software designed for model-based fitting of scattering data. Two different models were applied depending on the sample composition: the core-shell sphere model for the microemulsion (ME) droplets and menthol-containing samples, and the pearl-necklace model for selected formulations containing xanthan gum (XG), as detailed in Table 1 of the main text.

1. Core-Shell Sphere Model, applied to the samples ME, ME + menthol 0.1% w/w, and ME + menthol 1.0% w/w.

This model describes spherical particles composed of a core (the oil phase) and a concentric shell (composed by surfactant and cosurfactant), immersed in a solvent (the aqueous phase). According to this model, the form factor *P(q)* contribution to the scattering intensity *I(q)* can be described by the following equation:

$$P(q) = \frac{scale}{V_{total}} \times |F^2(q)| + background$$

in which the function *F(q)* is defined as:

$$F(q) = \frac{3}{V_s}[V_c(\rho_c - \rho_s)\frac{sin(qr_c) - qr_c\, cos(qr_c)}{(qr_c)^3} + V_s(\rho_s - \rho_{solv})\frac{sin(qr_s) - qr_s\, cos(qr_s)}{(qr_s)^3}]$$

In this function, V_s represents the volume of the whole particle (ME droplet in the current work), V_c the volume of oil core, ρ_c the scattering length density of the oil core, ρ_s the scattering length density of the shell formed by the hydrated polar headgroups of surfactant and cosurfactant, r_c the radius of the oil core and r_s the radius of the shell. ρ_{solv} is the scattering length density of the continuous aqueous phase. The scattering length densities for the different parts were estimated based on the SLD Calculator Tool available in the SasView software with the input parameters: molecular formula, molecular weight, density and X-ray wavelength.

2. Pearl-Necklace Model, applied to the samples men-XG-ME 1 to 5.

This model describes a chain composed of *n* spherical beads (pearls) of radius r_s, separated by flexible string segments. The form factor *P(q)* in this case is given by:

$$P(q) = \frac{scale}{V_{total}} \times \frac{\left(S_{ss}(q) + S_{rr}(q) + S_{rs}(q)\right)}{\left((M \times m_r) + (n \times m_s)\right)^2} + background$$

The term $S_{ss}(q)$ represents the intra-chain structure factor contribution arising from the scattering of the spherical pearls (the ME droplets), while the term $S_{rr}(q)$ is the intra-chain structure factor term that accounts for interference between the flexible "string" segments connecting the spherical pearls. The term $S_{rs}(q)$ is a mixed term that considers the correlations between the strings and the

pearls. As mentioned before, n denotes for the number of pearls, that is total number of spherical beads in the chain. M represents the number of connecting segments and is defined as $M = n$-1. The terms m_r and m_s are the scattering mass of a string segment and of a pearl, respectively, and can be calculated as:

$$m_r = \pi r_r^2 l \times (\rho_r - \rho_{solv})$$

$$m_s = \frac{4}{3}\pi r_s^3 \times (\rho_s - \rho_{solv})$$

in which r_r is the radius of the string, l is the edge-to-edge distance between pearls, and r_s is the radius of the pearls. The terms ρ are related to the scattering length densities of the strings (ρ_r), of the pearls (ρ_s), and of the solvent (ρ_{solv}) which were estimated using the SLD Calculator Tool as discussed above. Other details of the models used to fit SAXS data can be found in references 32-34 of the main text.

SAXS measurements

The incorporation of xanthan gum (XG) into the men-XG-ME2, ME4, and ME5 formulations resulted in the formation of polysaccharide chains associated with the droplet surfaces, leading to the appearance of cylindrical nanostructures commonly referred to as "pearl-necklace."

Figure S5. SAXS curves (open dots) for ME containing XG, along with the fits (black lines) to the pearl-necklace model. The data are shifted along the y-axis for better visualization.

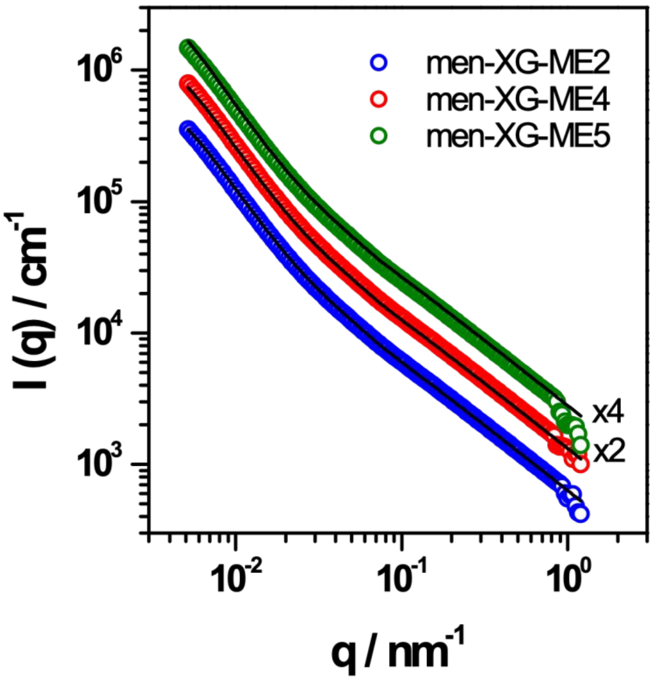

Experimental design and Response Surface Methodology (RSM) modeling

Below, we present the physicochemical characterization of the microemulsions containing X1 (xanthan gum) and X2 (menthol), along with detailed statistical analyses of rheological and texture properties, emphasizing the best-fitting mathematical models.

The application of the response surface method sheds a light on whether the independent variables (X1 or X2) or their interaction (X1:X2) are crucial for the observed response.

Table S1 shows the results of the measurements carried out with the formulations from the experimental design. The main effects and interactions were estimated from the measurements on the formulations. However, there was no significant effect of the two variables X1 and X2 for pH (Table S2). In addition, we summarize in Table S2 the regression analysis for all mathematical models performed. The detailed statistics can be found in the supplementary material (Supporting Information, Tables S3-S10), and in the following subsections, the obtained results are discussed in detail.

Consistency (TPA and rheology)

Consistency provides information about the viscosity of the fluid [1]. This parameter is important when a thicker layer of the formulation is applied to the skin, when the exposure time of the drug to the skin is prolonged, or when an occlusive layer must be formed to protect the skin. Consistency was determined in two different steps.

First, the consistency (g·s) of TPA was determined using the area under the positive curve for sample penetration [2,3]. TPA showed that only the independent variable X1 had significant effects on the consistency of the formulations of men-XG-ME, as shown in Fig. 4C. This quadratic model was significant ($p < 0.001$) (Table S2). Second, the consistency index ($Pa \cdot s^n$) obtained from rheological measurements was modeled using the Ostwald model. Although the bases of the two techniques are different, the mathematical modeling also showed that only the variable X1 had significant effects on the consistency – from rheological measurements – of the formulations of men- XG-ME (Fig. 4E). This linear model was significant ($p < 0.001$) (Table S2).

Our data showed that the polymer- and drug-free ME was a homogeneous, clear, colorless liquid. The addition of XG resulted in ME with increased consistency and thickness. Similar results were demonstrated by Djekic et al. (2016). They showed that higher concentrations of XG (0.25, 0.5, 0.75, and 1.00%) increased the thickness and consistency of ME [4]. In addition, increasing the concentration of XG led to an increase in viscosity, mucoadhesiveness, and formulation strength with econazole nitrate [5]. Gel strength was defined as a consistency-like parameter because strong gels can withstand much higher pressure than weak gels before washing out of the target area. Therefore, we hypothesize that XG can increase the consistency of the ME and maintain the structure of the ME and is a suitable matrix for the vehicle.

In rheology, the application of constant mechanical forces or shear rates to a sample allows the evaluation of rheological properties such as viscosity and elasticity. On the other hand, texture analyses mimic human sensory impressions and measure textural properties such as firmness, consistency, and adhesiveness [6–8]. Thus, differences in the results of these approaches are

highlighted due to the distinct nature of the mechanical solicitation, as exemplified by a product exhibiting high viscosity in rheology but is still perceived as soft in application. The importance of discussing such differences is emphasized, especially in studies aiming to understand the physical and sensory properties of products to enable the correlation between rheological characteristics and texture perception [1,7,8]. This understanding not only helps to explain inconsistencies between instrumental analyses and human sensory evaluations but also leads to improvements in product formulation to meet consumer expectations.

Adhesiveness (TPA)

Adhesiveness is defined as the work required to overcome the attractive forces between the surface of the sample and the surface of the probe with which the sample is in contact [9]. This parameter can provide an idea of the current residence time. The adhesiveness or viscosity index was defined using the area under curve of the negative TPA curve [2,3] . The independent variables X1 and X2 showed significant effects on the adhesiveness, as shown in the response surface diagram (Fig. S3A). The main effect graph and Pareto plot (Fig. S3B-E) show that increasing the variation of the concentration of X1, X2 and the interaction between the two parameters significantly affected the adhesiveness. From the linear interaction equation in Table 6 and Fig. 9, it can be seen that increasing the concentration of X1 and X2 has a positive effect on the adhesiveness. The data were fitted, and ANOVA tested the significance and adjustment of the model.

The use of polymers in pharmaceutical formulations as thickening agents aims to make the ME adhere longer to the administration site and thus reduce the frequency of application. Various polymers such as carbomers, polycarbophil, poly(ethylene oxide)-poly(propylene oxide)-poly(ethylene oxide), cellulose ethers, polysaccharides (e.g., xanthan gum, alginates) have been incorporated into liquid formulations to increase the adhesiveness of the evaluated systems [10,11]. The adhesive properties of xanthan gum have been used to develop controlled bioadhesive formulations and drug delivery systems [5][39]. Menthol also had a significant effect on adhesiveness. Menthol is a hydrophobic molecule with a high affinity for organic phases. Due to this property and the findings already discussed, we hypothesize that menthol migrates into the droplet and dissolves into oleic acid, increasing the droplet size. The increase in the volume of the organic phase due to the solubilization of menthol in oleic acid within the droplet promotes the increase in the adhesion of the system. Lipophilic components improve the ability of formulations to adhere to the skin [3]. Even though the highest increase here observed is about 30 nm. Kikuchi and co-workers (2021) showed that the adhesion strength increases with oil phase content in O/W emulsions [12].

Figure S6. Response surface plot for adhesiveness (g.s) obtained for the optimization of men-XG-ME formulations with two independent variables, xanthan gum (X1) and menthol (X2). **A**, Response surface plot. **B**, main effects of xanthan gum (X1). **C**, main effects of menthol (X2). **D**, Main effects of xanthan gum (X1) and menthol (X2). **E**, Pareto diagram showing the absolute effect of the factors xanthan gum (X1), menthol (X2), and interaction between xanthan gum and menthol (X1X2).

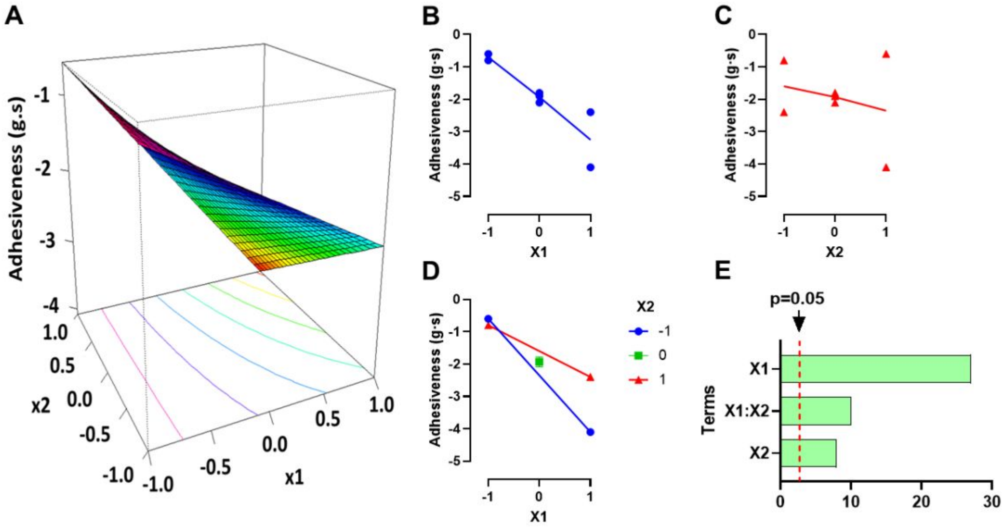

Cohesiveness (TPA)

The cohesiveness values were determined using the TPA curve. It is the maximum force of the negative curve in the TPA [3]. This parameter reflects the internal forces that must be overcome to spread the product on the skin surface [9]. The TPA showed that only the independent variable X1 had a significant effect on the cohesiveness of the men-XG-ME formulations (Fig. S4D). This quadratic model was significant ($p < 0.001$) (Table S2).

The addition of XG has been shown to significantly increase of the cohesiveness of hydrogel formulations [13]. Cohesiveness is an essential parameter for the development of formulations and is related to the resistance offered by the sample during application. The greater the cohesiveness parameter, the more intramolecular interactions and the greater the force required to remove the formulation from the tube or apply it to the skin surface, which can influence the residence time of the formulation during treatment [9].

Flow index (rheology)

The flow index is associated with the level of pseudoplasticity exhibited by the sample [1]. The independent variable XG (X1), but not menthol (X2), showed significant effects on the flow index, as can be seen in the main effects graph (Fig. S4F). All formulations exhibited a flow index below 1.0, indicating pseudoplastic behavior. From the graph, it can be seen that varying the concentration of X1 significantly affects the flow index of the men-XG-ME. The data were fitted to a linear model for X1 (Table S2). From the linear equation and Fig. S4F, it is evident that increasing the concentration of XG has a negative effect on the flow index.

The addition of XG to ME led to the formation of shear-thinning pseudoplastic systems with thixotropy. In such scenarios, the flow index decreases with increasing XG concentration, as shown in Figure S4F. The shear-thinning pseudoplastic flow behavior is typical for aqueous solutions and hydrogels containing XG. Studies in which XG was added to a ME in concentrations of 0.25% to 3% have shown that these also exhibit pseudoplastic shear-thinning rheological behavior [4,14–16].

Figure S7. Main effects of the experimental design of men-XG-ME formulations using two independent variables, xanthan gum (X1) and menthol (X2). **A**, conductivity (µS); **B**, firmness (g); **C**, consistency (g.s); **D**, cohesiveness (g); **E**, consistency index (Pa·s^n) and **F**, flow index.

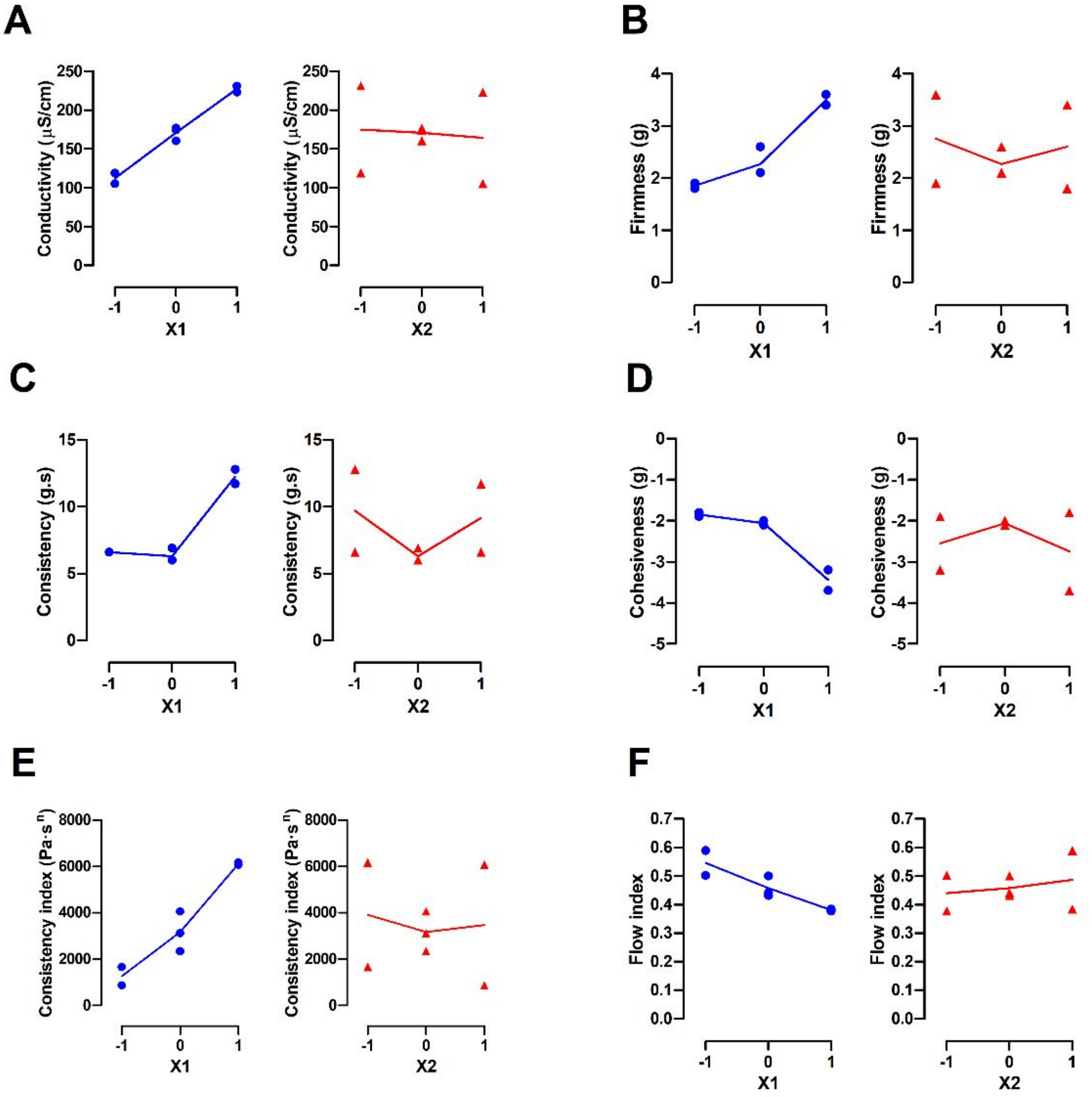

Table S1: Physicochemical characterization of the microemulsions in the presence of X1 (xanthan gum) and X2 (menthol).

men-XG-ME	X1	X2	pH	Conductivity (µS/cm)	Firmness (g)	Consistency (g·s)	Adhesiveness (g·s)	Cohesiveness (g)	Consistency index (Pa·s^n)	Flow index
**1**	-1	-1	4.6	119.0	1.9	6.6	-0.8	-1.9	1663.4	0.5021
**2**	1	-1	4.5	231.0	3.6	12.8	-2.4	-3.2	6160.9	0.3781
**3**	-1	1	4.6	105.0	1.8	6.6	-0.6	-1.8	870.43	0.5897
**4**	1	1	4.6	223.0	3.4	11.7	-4.1	-3.7	6073.8	0.3842
**5**	0	0	4.6	160.3	2.6	6.9	-2.1	-2.1	4059.4	0.4321
**6**	0	0	4.5	176.8	2.1	6.0	-1.9	-2.0	3120.8	0.4412
**7**	0	0	4.5	174.3	2.1	6.0	-1.8	-2.1	2342.0	0.5001

Table S2: Summary of regression analysis for responses X_1 (xanthan gum) and X_2 (menthol).

Effect	Model	R^2	Adjusted R^2	Residuals	p-value
**Conductivity (µS)**	$Y= 169.914 +57.500 \cdot X1$	0.9786	0.9743	7.613	0.000023
**Firmness (g)**	$Y= 2.26667+ 0.82500 \cdot X1 + 0.40833 \cdot X1^2$	0.9401	0.9102	0.191	0.003588
**Consistency (g.s)**	$Y= 6.30000 + 2.82500 \cdot X1 + 3.12500 \cdot X1^2$	0.9770	0.9655	1.145	0.000529
**Adhesiveness (g.s)**	$Y= -1.95714 - 1.27500 \cdot X1 - 0.37500 \cdot X2 - 0.47500 \cdot X1X2)$	0.9938	0.9876	0.129	0.000825
**Cohesiveness (g)**	$Y= -1.76667 - 1.22500 \cdot X1 - 0.45833 \cdot X1^2$	0.9505	0.9257	0.3317	0.002455
**Consistency index ($Pa\cdot s^n$)**	$Y= 3470 + 2425 \cdot X1$	0.9125	0.8949	671.9	0.000795
**Flow index**	$Y= 0.46107 - 0.08237 \cdot X1$	0.8035	0.7642	0.03643	0.006272

Rheology and texture analyzes

Table S3. The detailed statistics from pH.

pH					
**Coefficients**	**Estimate**	**Std. Error**	**t value**	**Pr(>|t|)**	**Significance level**
Intercept	4.54500	0.02879	157.854	0.0000401	***
x1	-0.01825	0.02493	-0.732	0.540	NS
x2	-0.00025	0.02493	-0.010	0.993	NS
x1, x2	0.02525	0.02493	1.013	0.418	NS
$x1^2$	0.02225	0.03809	0.584	0.618	NS
$x2^2$	NA	NA	NA	NA	NS

Note: ***p<0.001; NS: Not Significant

Table S4. The detailed statistics from conductivity.

Conductivity (µS/cm)					
**Coefficients**	**Estimate**	**Std. Error**	**t value**	**Pr(>|t|)**	**Significance level**
Intercept	169.914	2.877	59.05	2.63e-08	***
x1	57.500	3.806	15.11	2.30e-05	***

Note: ***p<0.001

Table S5. The detailed statistics from firmness (TPA).

Firmness (g)					
**Coefficients**	**Estimate**	**Std. Error**	**t value**	**Pr(>|t|)**	**Significance level**
Intercept	2.26667	0.12638	17.9351	5.68e-05	***
x1	0.82500	0.10945	7.5377	0.001659	**
$x1^2$	0.40833	0.16719	2.4424	0.071026	·

Note: ***p<0.001; **p<0.01; ·p<0.1

Table S6. The detailed statistics from consistency (TPA).

Consistency (g·s)					
**Coefficients**	**Estimate**	**Std. Error**	**t value**	**Pr(>|t|)**	**Significance level**
Intercept	6.30000	0.30890	20.3952	3.41e-05	***
x1	2.82500	0.26751	10.5603	0.0004549	***
$x1^2$	3.12500	0.40863	7.6475	0.0015708	**

Note: ***p<0.001; **p<0.01

Table S7. The detailed statistics from adhesiveness (TPA).

Adhesiveness (g·s)					
**Coefficients**	**Estimate**	**Std. Error**	**t value**	**Pr(>|t|)**	**Significance level**
Intercept	-1.95714	0.04862	-40.254	3.37e-05	***
x1	-1.27500	0.06432	-19.823	0.000281	***
x2	-0.37500	0.06432	-5.830	0.010051	*
x1:x2	-0.47500	0.06432	-7.385	0.005134	**

Note: ***p<0.001; **p<0.01; *p<0.05

Table S8. The detailed statistics from cohesiveness (TPA).

Cohesiveness (g)					
**Coefficients**	**Estimate**	**Std. Error**	**t value**	**Pr(>|t|)**	**Significance level**
Intercept	-1.76667	0.16625	-10.6266	0.000444	***
x1	-1.22500	0.14398	-8.5083	0.001047	**
$x1^2$	-0.45833	0.21993	-2.0840	0.105542	

Note: ***p<0.001; **p<0.01

Table S9. The detailed statistics from consistency index (rheology).

Consistency index (Pa·sn)					
**Coefficients**	**Estimate**	**Std. Error**	**t value**	**Pr(>|t|)**	**Significance level**
Intercept	3470	254	13.664	3.77e-05	***
x1	2425	336	7.219	0.000795	***

Note: ***p<0.001

Table S10. The detailed statistics from flow index (rheology).

Flow index					
**Coefficients**	**Estimate**	**Std. Error**	**t value**	**Pr(>|t|)**	**Significance level**
Intercept	0.46107	0.01377	33.482	4.47e-07	***
x1	-0.08237	0.01822	-4.522	0.00627	**

Note: ***p<0.001; **p<0.01

Rheology and TPA correlation

Relationship between rheological parameters (consistency index *K* and flow index *n*) and Texture Profile Analysis (TPA) parameters (firmness, consistency, adhesiveness, and cohesiveness), we tested five different regression models: linear, polynomial, logarithmic, power, and exponential (Table S11).

Table S11. Comparative regression analysis of the effects of X1 (xanthan gum) and X2 (menthol) on analytical responses using multiple mathematical models.

**Consistency index ($Pa·s^n$) vs Firmness (g)**		
**Mathematical models**	**Equation**	**R^2**
Linear	$y = 0.0003x + 1.308$	0.9507
Polynomial	$y = 5E\text{-}08x^2 - 3E\text{-}06x + 1.7767$	0.9887
Logarithmic	$y = 0.9093\ln(x) - 4.7405$	0.7843
Power	$y = 0.1402x^{0.3576}$	0.8613
Exponential	$y = 1.5261e^{0.0001x}$	0.9780

**Consistency index ($Pa·s^n$) vs Consistency (g·s)**		
**Mathematical models**	**Equation**	**R^2**
Linear	$y = 0.0012x + 3.9103$	0.7495
Polynomial	$y = 5E\text{-}07x^2 - 0.0022x + 8.5161$	0.9848
Logarithmic	$y = 2.877\ln(x) - 14.822$	0.5044
Power	$y = 0.6186x^{0.3168}$	0.5901
Exponential	$y = 4.8532e^{0.0001x}$	0.8241

**Consistency index ($Pa·s^n$) vs Adhesiveness (g·s)**		
**Mathematical models**	**Equation**	**R^2**
Linear	$y = -0.0005x - 0.2518$	0.7767
Polynomial	$y = 3E\text{-}08x^2 - 0.0007x + 0.0124$	0.7815
Logarithmic	$y = -1.407\ln(x) + 9.2496$	0.7499
Power	N/A	
Exponential	N/A	

Consistency index ($Pa \cdot s^n$) vs Cohesiveness (g)		
**Mathematical models**	**Equation**	**R^2**
Linear	$-0.0005x - 0.2967$	0.9594
Polynomial	$y = -4E\text{-}08x^2 - 0.0002x - 0.7085$	0.9734
Logarithmic	$y = -1.353\ln(x) + 8.7421$	0.8296
Power	N/A	
Exponential	N/A	

Flow index vs Firmness (g)		
**Mathematical models**	**Equation**	**R^2**
Linear	$y = -8.457x + 6.399$	0.7550
Polynomial	$y = 62.343x^2 - 68.159x + 20.372$	0.9450
Logarithmic	$y = -4.117\ln(x) - 0.7325$	0.8068
Power	$y = 0.6856x^{-1.604}$	0.8756
Exponential	$y = 11.124e^{-3.312x}$	0.8325

Flow index vs Consistency ($g \cdot s$)		
**Mathematical models**	**Equation**	**R^2**
Linear	$y = -27.43x + 20.73$	0.5104
Polynomial	$y = 349.06x^2 - 361.71x + 98.968$	0.8932
Logarithmic	$y = -13.7\ln(x) - 2.6733$	0.5742
Power	$y = 2.3532x^{-1.511}$	0.6666
Exponential	$y = 31.061e^{-3.022x}$	0.5998

Flow index vs Adhesiveness (g·s)		
**Mathematical models**	**Equation**	**R^2**
Linear	$y = 12.81x - 7.863$	0.6912
Polynomial	$y = -41.695x^2 + 52.737x - 17.208$	0.7251
Logarithmic	$y = 6.1099\ln(x) + 2.8405$	0.7094
Power	N/A	
Exponential	N/A	

Flow index vs Cohesiveness (g)		
**Mathematical models**	**Equation**	**R^2**
Linear	$y = 12.58x - 7.831$	0.7992
Polynomial	$y = -76.175x^2 + 85.533x - 24.904$	0.9348
Logarithmic	$y = 6.0864\ln(x) + 2.7506$	0.8430
Power	N/A	
Exponential	N/A	

Final remarks:

These findings contribute to the development of predictive models for the behavior of topical formulations, aiding in the prediction of their interaction with the skin and supporting the design of future topical drug delivery systems. The mathematical models developed in this study offer a foundation for generating prototypes with customized properties that maximize the pharmacological potential of menthol.

In summary, this study not only uncovers novel insights into the formulation of menthol-loaded microemulsion but also sets the stage for future innovations in drug delivery systems. The discovery of "pearl necklace" structures induced by XG opens new avenues for advanced dermatological treatments and targeted therapies, benefiting from enhanced solubility and stability. Additionally, the integration of sophisticated analytical techniques establishes a new standard for investigating microstructures in pharmaceutical formulations, with implications for improving both therapeutic efficacy and the robustness of modern pharmaceutical products. Ultimately, this research contributes meaningfully to the advancement of scientific knowledge and inspires further translational and applied research, to improve patient outcomes through pharmaceutical innovation.

Reference

(1) César, F. C. S.; Maia Campos, P. M. B. G. Influence of Vegetable Oils in the Rheology, Texture Profile and Sensory Properties of Cosmetic Formulations Based on Organogel. *Int. J. Cosmet. Sci.* **2020**, *42* (5), 494–500. https://doi.org/10.1111/ics.12654.

(2) Tai, A.; Bianchini, R.; Jachowicz, J. Texture Analysis of Cosmetic/Pharmaceutical Raw Materials and Formulations. *Int. J. Cosmet. Sci.* **2014**, *36* (4), 291–304. https://doi.org/10.1111/ics.12125.

(3) Calixto, L. S.; Maia Campos, P. M. B. G. Physical-Mechanical Characterization of Cosmetic Formulations and Correlation between Instrumental Measurements and Sensorial Properties. *Int. J. Cosmet. Sci.* **2017**, *39* (5), 527–534. https://doi.org/10.1111/ics.12406.

(4) Djekic, L.; Martinovic, M.; Stepanović-Petrović, R.; Micov, A.; Tomić, M.; Primorac, M. Formulation of Hydrogel-Thickened Nonionic Microemulsions with Enhanced Percutaneous Delivery of Ibuprofen Assessed in Vivo in Rats. *Eur. J. Pharm. Sci.* **2016**, *92*, 255–265. https://doi.org/10.1016/j.ejps.2016.05.005.

(5) Evelyn, D.; Wooi, C. C.; Kumar, J. R.; Muralidharan, S.; Dhanaraj, S. A. Development and Evaluation of Microemulsion Based Gel (MBGs) Containing Econazole Nitrate for Nail Fungal Infection. *J. Pharm. Res.* **2012**, *5* (4), 2385–2390.

(6) Jones, D. S.; Lawlor, M. S.; Woolfson, A. D. Examination of the Flow Rheological and Textural Properties of Polymer Gels Composed of Poly(Methylvinylether-Co-Maleic Anhydride) and Poly(Vinylpyrrolidone): Rheological and Mathematical Interpretation of Textural Parameters. *J. Pharm. Sci.* **2002**, *91* (9), 2090–2101. https://doi.org/10.1002/jps.10195.

(7) Estanqueiro, M.; Amaral, M. H.; Sousa Lobo, J. M. Comparison between Sensory and Instrumental Characterization of Topical Formulations: Impact of Thickening Agents. *Int. J. Cosmet. Sci.* **2016**, *38* (4), 389–398. https://doi.org/10.1111/ics.12302.

(8) Calixto, L. S.; Infante, V. H. P.; Maia Campos, P. M. B. G. Design and Characterization of Topical Formulations: Correlations Between Instrumental and Sensorial Measurements. *AAPS PharmSciTech* **2018**, *19* (4), 1512–1519. https://doi.org/10.1208/s12249-018-0960-0.

(9) Froelich, A.; Osmałek, T.; Kunstman, P.; Roszak, R.; Białas, W. Rheological and Textural Properties of Microemulsion-Based Polymer Gels with Indomethacin. *Drug Dev. Ind. Pharm.* **2016**, *42* (6), 854–861. https://doi.org/10.3109/03639045.2015.1066799.

(10) Djekic, L.; Martinović, M.; Dobričić, V.; Čalija, B.; Medarević, Đ.; Primorac, M. Comparison of the Effect of Bioadhesive Polymers on Stability and Drug Release Kinetics of Biocompatible Hydrogels for Topical Application of Ibuprofen. *J. Pharm. Sci.* **2019**, *108* (3), 1326–1333. https://doi.org/10.1016/j.xphs.2018.10.054.

(11) Collaud, S.; Warloe, T.; Jordan, O.; Gurny, R.; Lange, N. Clinical Evaluation of Bioadhesive Hydrogels for Topical Delivery of Hexylaminolevulinate to Barrett's Esophagus. *J. Control. Release* **2007**, *123* (3), 203–210. https://doi.org/10.1016/j.jconrel.2007.08.015.

(12) Kikuchi, K.; Mayama, H.; Nonomura, Y. Nonlinear Friction Dynamics of Oil-in-Water and Water-in-Oil Emulsions on Hydrogel Surfaces. *Langmuir* **2021**, *37* (26), 8045–8052. https://doi.org/10.1021/acs.langmuir.1c01339.

(13) Alves, A.; Miguel, S. P.; Araujo, A. R. T. S.; de Jesús Valle, M. J.; Sánchez Navarro, A.;

Correia, I. J.; Ribeiro, M. P.; Coutinho, P. Xanthan Gum–Konjac Glucomannan Blend Hydrogel for Wound Healing. *Polymers (Basel).* **2020**, *12* (1), 99. https://doi.org/10.3390/polym12010099.
(14) Singhvi, G.; Hans, N.; Shiva, N.; Kumar Dubey, S. Xanthan Gum in Drug Delivery Applications. In *Natural Polysaccharides in Drug Delivery and Biomedical Applications*; Elsevier, 2019; pp 121–144. https://doi.org/10.1016/B978-0-12-817055-7.00005-4.
(15) Jaya Raja Kumar, K.; Muralidharan, S.; Dhanaraj, S. A. Anti-Fungal Activity of Microemulsion Based Fluconazole Gel for Onychomycosis against Aspergillus Niger. *Int. J. Pharm. Pharm. Sci.* **2013**, *5* (1), 96–102.
(16) Rao, M.; Sukre, G.; Aghav, S.; Kumar, M. Optimization of Metronidazole Emulgel. *J. Pharm.* **2013**, *2013*, 1–9. https://doi.org/10.1155/2013/501082.
